# Avoiding Conflict: When Speaker Coordination Does Not Require Conceptual Agreement

**DOI:** 10.3389/frai.2020.523920

**Published:** 2021-01-27

**Authors:** Alexandre Kabbach, Aurélie Herbelot

**Affiliations:** ^1^ Department of Linguistics, University of Geneva, Geneva, Switzerland; ^2^ Center for Mind/Brain Sciences, University of Trento, Trento, Italy; ^3^ Department of Information Engineering and Computer Science, University of Trento, Trento, Italy

**Keywords:** communication, coordination, alignment, conceptual variability, distributional semantic models, similarity, socialization hypothesis

## Abstract

In this paper we discuss the *socialization hypothesis*—the idea that speakers of the same (linguistic) community should share similar concepts given that they are exposed to similar environments and operate in highly-coordinated social contexts—and challenge the fact that it is assumed to constitute a prerequisite to successful communication. We do so using *distributional semantic models* of meaning (DSMs) which create lexical representations via latent aggregation of co-occurrence information between words and contexts. We argue that DSMs constitute particularly adequate tools for exploring the socialization hypothesis given that 1) they provide full control over the notion of background environment, formally characterized as the training corpus from which distributional information is aggregated; and 2) their geometric structure allows for exploiting alignment-based similarity metrics to measure inter-subject alignment over an entire semantic space, rather than a set of limited entries. We propose to model *coordination* between two different DSMs trained on two distinct corpora as *dimensionality selection* over a dense matrix obtained via Singular Value Decomposition This approximates an ad-hoc coordination scenario between two speakers as the attempt to align their similarity ratings on a set of word pairs. Our results underline the specific way in which linguistic information is spread across singular vectors, and highlight the need to distinguish *agreement* from mere *compatibility* in alignment-based notions of conceptual similarity. Indeed, we show that *compatibility emerges from idiosyncrasy* so that the unique and distinctive aspects of speakers’ background experiences can actually facilitate—rather than impede—coordination and communication between them. We conclude that the socialization hypothesis may constitute an unnecessary prerequisite to successful communication and that, all things considered, communication is probably best formalized as the cooperative act of *avoiding conflict*, rather than maximizing agreement.

## 1 Introduction

Psychological approaches to semantic and conceptual knowledge rely on intertwined yet distinct notions of *concepts* and *words* (Malt et al., 2015; Malt, 2019): concepts are “the building blocks of thought” taken to be crucial to cognition at large (Margolis and Laurence, 2019), while words are “the smallest linguistic expressions conventionally associated with non-compositional meaning […] which can be articulated in isolation to convey semantic content” (Gasparri and Marconi, 2019). Those psychological approaches—also referred to as *cognitivist* or *subjectivist* (Gärdenfors, 2014; Barsalou, 2017; Pelletier, 2017)—assume concepts, unlike words, to be *private* mental entities, which poses a major challenge for communication, for how could two speakers communicate if the words they utter do not refer to identical concepts? (Fodor, 1977; Pelletier, 2017).

The solution to this conundrum, we are told, lays in the inherently social nature of the lexical acquisition process (Clark, 1996; Murphy, 2002; Barsalou, 2017) for if children do acquire lexical items by matching new words to previously learned concepts (e.g., Bloom, 2000) they do not do so randomly: they learn through socialization which concepts go with which words, so that the internal mental representations associated with words are shaped by many years of interactions with other speakers of the same (linguistic) community. As a result, speakers of the same community relate words to very *similar* concepts (Murphy, 2002, p. 391). The *socialization hypothesis*—as we propose to name it—therefore postulates that speakers of the same community *should* share similar concepts given that they are exposed to similar environments and operate in highly-coordinated social contexts (see Section 2.1).

Yet, conceptual similarity remains *hard* to validate experimentally, and is more often than desired a matter of seeing the glass as half full: speakers never significantly disagree on their judgments of similarity, but never totally agree either (see Section 2.2). Meanwhile, recent work in cognitive science has attempted to come to term with the idea that concepts may vary widely across individuals, some even suggesting that it may not necessarily represent an obstacle to communication, as what matters ultimately is that speakers coordinate *during* conversation and *align* their conceptual representations on aspects relevant to the situation under discussion (see Section 2.3).

Yet again, this notion of *alignment* remains dubious as it is often relaxed to mere *similarity* or *sufficient overlap*. But what does it *mean* for two concepts to be similar? And how much similarity is *enough* for successful communication? In fact, alignment-based similarity appears more often than not to be a matter of overall *compatibility* rather than strict *agreement*: being highly tolerant to variability, it can potentially settle for minimal overlap so that speakers holding marginally identical conceptual representations can still be assumed to understand one another. But if *anything goes*, then this notion of similarity becomes rather devoid of content and pretty much useless for assessing the pertinence of the socialization hypothesis.

As always, the devil is in the details. For indeed the socialization hypothesis focuses on conceptual *spaces* and as such pertains to the *whole structure* rather than the *superficial parts*. After all, the notion of conceptual variability considered so far remains superficial in as much as it is only observed through the lens of limited behavioral response patterns in humans. And since *superficial variability does not preclude latent structural similarity*, conceptual spaces could still very well be aligned despite the apparent variability, provided the adequate characterization of alignment (see Section 2.4). Additional methodological challenges still remain in order to validate the socialization hypothesis, for 1) it is never possible to gain full access over speakers’ background experiences which presumably condition the formation of their respective conceptual spaces; and 2) it is in practice never possible to test human subjects on their entire lexicons, let alone conceptual spaces, in order to guarantee the robustness of the observed experimental results.

To overcome parts of those methodological challenges, we propose in this work to rely on distributional semantic models of lexical meaning (DSMs) which create vector representations for words via latent aggregation of co-occurrences between words and contexts (see Section 3). We argue that those models prove particularly suited for assessing the validity of the socialization hypothesis, given that 1) they provide full control over speakers’ background experiences, formalized experimentally as the training corpus from which distributional information is aggregated; 2) their geometric structure allows for exploiting alignment-based similarity metrics to measure inter-subject alignment, and do so over an entire semantic space rather than a set of limited entries, thereby overcoming the experimental shortcomings of testing on human subjects; and 3) their overall generation pipeline parallels humans’ conceptual processing in a cognitively plausible fashion.

Following the core assumptions underpinning the socialization hypothesis stated above, we propose to distinguish within our model *background experience* from *active coordination*. On the one hand, we control for background experience by varying the data fed to the DSM. On the other hand, we implement *active coordination* by modifying the standard DSM pipeline, which normally includes a dimensionality reduction step involving the top singular vectors of a Singular Value Decomposition (SVD). Specifically, we replace the variance-preservation bias by an explicit coordination bias, sampling the set of *d* singular vectors which maximize the correlation with a particular similarity dataset (see Section 4.1). Thereby, we approximate an ad-hoc coordination scenario between two speakers as the attempt to align their similarity ratings on a set of word pairs. We then propose to quantify structural alignment between two DSMs as the residual error between their two matrices, measured after having put their elements in correspondence with one-another (see Section 4.2).

Using the above methodology, the paper makes three contributions. First, we show that *no variance-preservation bias means better superficial alignment*. Indeed, we show that replacing the variance-preservation bias by an explicit sampling bias leads to near-systematic improvements on various lexical similarity datasets. We show in addition that this result is fundamentally grounded in the fact that *different dimensions in the SVD encode different semantic phenomena*, so that DSMs can actually capture a collection of possible meaning spaces from the same set of data, rather than a single one (see Section 5.1).

Second, we show that *better superficial alignment does not mean better structural alignment*. Although alignment is arguably a complex and multifaceted process, we show that, when considered from the point of view of our specific characterization, the systematicity of the relation between superficial and structural alignment does not hold (see Section 5.2).

Third, we show that conceptual spaces generated from different background experiences can be aligned in different ways, and that the aforementioned considerations over *alignment* and *compatibility* extend from conceptual *representations* to conceptual *spaces*. Indeed, we show that DSMs can be aligned by sampling pairs of singular vectors which highly correlate with one another, but also very often by sampling singular vectors that do not correlate but nonetheless increase the structural similarity between the two modeled conceptual spaces (see Section 5.3). A deeper investigation of this effect suggests that *compatibility emerges from idiosyncrasy*, so that the unique and distinctive aspects of speakers’ background experiences can actually facilitate—rather than impede—coordination and communication between them (see Section 6).

We conclude that the socialization hypothesis may constitute an unnecessary prerequisite to successful communication and that, all things considered, communication is probably best formalized as the cooperative act of *avoiding conflict*, rather than maximizing agreement.

## 2 Conceptual Variability and the Socialization Hypothesis

### 2.1 The Socialization Hypothesis: Review and Overview

The primary observation underpinning the socialization hypothesis is that conceptual acquisition precedes lexical acquisition, so that children first acquire concepts before learning to map them to corresponding lexical labels (Clark, 1983; Mervis, 1987; Merriman et al., 1991; Bloom, 2000). The key idea behind the hypothesis is then to consider that the acquisition of this conceptual-to-lexical mapping is not random but rather heavily constrained, in that it takes place in a highly coordinated social context, so that speakers of the same community end up assigning similar concepts to the same words. Phrased along those lines, the hypothesis can be found in (Murphy, 2002, p. 391):

[…] people do not associate any old concept to a word. Instead, they learn through socialization which concepts go with which words. So, as a child, you learned that dog refers to a certain kind of animal. If you first developed the hypothesis that dog refers to any four-legged mammal, you would soon find yourself miscommunicating with people. Theywould not understand you when you referred to a sheep as dog, and you would not understand them when they said that all dogs bark, and so on. Thus, there is a social process of converging on meaning that is an important (and neglected) aspect of language […]

However, the socialization hypothesis extends beyond the conceptual-to-lexical mapping itself: since human beings should have similar cognitive systems and evolve in similar environments overall, they should end up sharing similar *conceptual spaces* (Barsalou, 2017, p. 15):

[…] different individuals have similar bodies, brains, and cognitive systems; they live in similar physical environments; they operate in highly-coordinated social contexts. As a result, different individuals acquire similar distributed networks for a given concept over the course of development. Within a particular social group or culture, different individuals’ networks are likely to be highly similar, given similar coordinated experiences with many shared exemplars. Even across different cultures, these networks are likely to be highly similar, given that all humans have similar bodies, brains, and cognitive systems, operating in similar physical and social environments.

In both Murphy’s and Barsalou’s formulations of the hypothesis we find the idea that there are both individual and collective—cognitive and social—processes at play in both conceptual and lexical acquisition, as well as linguistic communication as a whole. The underlying idea is that people *cooperate* with one another when they use language (Austin, 1962; Grice, 1975) and perform what Clark (1996) has called *joint actions* on top of individual actions, so that they coordinate with one another in order to converge to some *common ground* (Clark, 1992; Clark, 1996). This notion of common ground (see also Stalnaker, 2002; Stalnaker, 2014) encompasses notions of *common knowledge* (Lewis, 1969), *mutual knowledge* or *belief* (Schiffer, 1972) and *joint knowledge* (McCarthy and Lifschitz, 1989) and covers whatever knowledge or beliefs speakers of the same (linguistic and/or cultural) community may share. It also includes what Gärdenfors (2014) refers to as *third-order intersubjectivity*: not only what I know, but also what I assume you know and what I assume you know that I know. Overall, the general idea put forth by Clark (1996) is that the more time people spend together, the larger their common ground; an idea which we can re-interpret in light of the socialization hypothesis as *shared experiences entail shared conceptual spaces*.

But coordination is also a process which takes place at the lexical level so that speakers can settle for a particular word meaning, a phenomenon that Clark (1992) has called *entrainment*.^1^ As such, and in as much as the socialization hypothesis can be said to presuppose meaning to derive from *convention*, one can trace its foundational considerations to Plato’s *Cratylus* (Cooper, 1997) and its discussion on the essence of meaning. According to Rescorla (2019), there is now a wide consensus in philosophy to stand with Hermogenes against Cratylus in considering that language at large is conventional, in that the association between a word and its referent is arbitrary and driven by convention rather than intrinsic to the nature of words. Conventional views of meaning have given rise to a very rich literature since the *signaling games* of Lewis (1969) which have proposed a formal characterization of the phenomenon of semantic convergence, grounded in Gricean pragmatics and the idea that meaning emerges from active coordination between speakers’ communicative intentions and hearers’ expectations (Grice, 1969).

Conventional views of meaning do not preclude however the semantics of a word to vary across time, or even across utterances. Cruse for instance, has argued that the meaning of a word changed to some extent at each of its occurrences—what he has called *context modulation* (Cruse, 1986, p. 52). Barker (2002) further observed that utterances could shift the meaning of a predicate, and those considerations have led several researchers to propose the idea of the existence of a *core* meaning for each word sense, core meaning potentially pragmatically modulated at each utterance (Lasersohn, 1999; Recanati, 2004; Wilson and Carston, 2007). Such considerations extend to concepts at large and the question of whether or not they have *cores* themselves (see Barsalou, 2017, for an overview). Indeed, several proposals have been made to argue against the notion of conceptual core and for the idea that concepts are, in part of in full, context-dependent (Evans, 2009; Connell and Lynott, 2014; Casasanto and Lupyan, 2015). This argument is partly supported by empirical evidence showing that not all conceptual information, even what could be considered central one, is automatically activated across context (Kiefer et al., 2012; Gawronski and Cesario, 2013; Lebois et al., 2015).

However, and despite the above consideration over conceptual variability, the socialization hypothesis remains grounded in the idea that identicity of concepts across speakers is not necessary for successful communication: sufficient conceptual *overlap* or *similarity* suffice. This idea can be found as early as (Humboldt, 1836/1988, p.152), when stating that:

Men do not understand one another […] by mutually occasioning one another to produce exactly and completely the same concept; they do it by touching in one another the same link in the chain of their sensory ideas and internal conceptualizations, by striking the same note on their mental instrument, whereupon matching but not identical concepts are engendered in each.

Relaxing the constraint over conceptual identicity across subjects remains nonetheless problematic, for it pushes the burden of proof over to the notion of similarity: what does it mean for two concepts to be *similar*? And how much similarity is *enough* for successful communication? (see, e.g., Connell and Lynott, 2014, p. 400). As we will see in the following section, unequivocally aligning similarity judgments is difficult to achieve across human subjects, and the proper characterization of similarity remains both a theoretical and an experimental challenge, so that the question of whether or not two speakers hold similar conceptual spaces is sometimes left to seeing the glass as half full.

### 2.2 Conceptual Similarity: An Experimental Challenge

What does it *mean* to *hold* a concept? As a first approximation, Murphy (2002) proposes to assimilate conceptual knowledge to lexical knowledge, although it has been convincingly argued that words do not begin to capture the richness of their underlying conceptual representations (Landau et al., 2010; Wolff and Malt, 2010; Gleitman and Papafragou, 2012). Marconi (1997) proposes to further distinguish within lexical knowledge the notion of *inferential* competence—the ability to *name* objects—from the notion of *referential* competence—the ability to *refer* to objects. This distinction is supported by empirical evidence from neuroscience showing that certain brain pathologies may affect one competence while leaving the other intact (Warrington, 1975; Heilman et al., 1976; Kemmerer et al., 2012; Pandey and Heilman, 2014). Marconi (1997) takes it for granted that lexical competence may vary widely across speakers of the same language, for language reflects what Putnam (1975) has called the *division of linguistic labor* which derives from the division of *non*-linguistic labor. That is, knowledge effects entailed by differences in expertise on a given domain may translate as differences in lexical knowledge across speakers. Yet, Marconi still assumes that certain parts of the lexicon will remain preserved from the interference of specialized knowledge, so that lexical competence for a certain number of words can be considered reasonably identical across speakers. He takes the word *spoon* to be one such example (Marconi, 1997, p. 57), and yet Labov (1973) showed in his seminal work on the semantics of tableware items that denotation for words such as *mug*, *cup*, *bowl* and *vase* could vary widely across individuals when modifying objects properties such as *width*, *depth*, *content* or even *presence or absence of a handle*. Labov’s study illustrates what has since been confirmed over and over experimentally, and what Pelletier summarizes as the fact that “different subjects give individually different results on the many tasks about meaning that have been administered over the decades in cognitive psychology” (Pelletier, 2017, p. 74). Indeed, psychological experiments on lexical similarity—which typically ask subjects to grade lists of word pairs on a ten-point scale, or triangular arrays of words by choosing among a pair of word the most similar to a referent word (Hutchinson and Lockhead, 1977)—exhibit mixed levels of agreement across subjects: from 0.44 to 0.63 on word pairs and from 0.45 to 0.66 on triangular arrays depending on the categories being tested (e.g., fruits or birds; see Hutchinson and Lockhead, 1977, p. 667).

Those results could be considered artifactual of experimental setups artificially decontextualizing lexical items by presenting them in isolation and without sentential context—potentially ignoring thereby the effect of *context modulation* (see Section 2.1). And indeed Anderson and Ortony (1975) confirmed experimentally that subjects modulate the meaning of a word at least based on the sentence in which it occurs. Murphy and Andrew (1993) even showed that subjects could change their judgments over synonyms and antonyms depending on the presented word pairs. Nonetheless, even experiments which do try to evaluate human similarity judgments in heavily constrained contextual setups exhibit non-trivial inter-speaker variability: in their study comparing lexical expectations across individuals, Federmeier and Kutas (1999) presented subjects with clozed sentence pairs such as *They wanted to make the hotel look more like a tropical resort. So along the driveway, they planted rows of* … and three target words comprising an expected exemplar (e.g., *palms* for the above example), an unexpected exemplar of the same category (e.g., *pines*) and an unexpected exemplar of a different category. Expectations regarding missing words were first evaluated as clozed probabilities computed by asking a set of subjects to select the best target candidate given the presented context, but only averaged at 0.74 while ranging from 0.17 to 1 depending on tested items. Other lexical substitution experiments performed on humans exhibit similarly low agreement levels across subjects: 0.28 for McCarthy and Navigli (2009) and as low as 0.19 and 0.16 for Kremer et al. (2014) and Sinha and Mihalcea (2014).

Could such relatively moderate levels of agreement constitute mere byproducts of the unreliability of introspective judgment? The question is not quite settled: Federmeier and Kutas (1999) did attempt to analyze the distribution of N400 across subjects—a negative-going potential peaking around 400ms after stimulus onset which often indicates semantic anomaly or an unexpected event. Yet, and although they did find slight differences in N400 patterns across subjects, they blamed the intrinsic variation of brainwaves across individuals and did not investigate further given the relatively small size ~ (6) of their sample of participants.

Of course, one could also say that lab experiments operatenecessary methodological approximations which lead to unrealistic language usage setups that do not, all things considered, invalidate the socialization hypothesis: communication is not a clozed test, let alone a lexical similarity task. Lexical variability at the word level, even if attested experimentally, does not preclude conceptual similarity to be validated when language takes places in a realistic, articulated, and coordinated communication setting. Words are seldom if ever used in isolation to refer to their underlying conceptual representations, and vice versa. Yet, inter-speaker variations in concept-to-word mappings led to very concrete problems when attempting to design verb-mediated computer interfaces in the 1990s: Furnas et al. (1987) for instance showed that agreement on (computer-) function-to-word mapping ranged from 0.07 to 0.18, and agreement on word-to-function mapping remained at 0.15 (see also Brennan, 1998). In other words, subjects barely used the same word to refer to the same function/concept, or thought of identical functions/concepts when using the same word, rendering verb-mediated computer interfaces practically unusable.

The notion of (conceptual and/or semantic) *similarity* itself is a challenge: it varies with experience, knowledge, expertise or even (linguistic) context (see Medin et al., 1993; Goldstone and Son, 2012, for an overview). Its theoretical foundations are somehow shaky, for A is always similar to B *with respect to something* (Goodman, 1972). Therefore, it pushes yet again the burden of proof over to modeling considerations on the notion of *context*, especially as similarity judgments remain sensitive to *tasks* (Murphy and Medin, 1985) and *instructions* (Melara et al., 1992).

We could still acknowledge the ubiquity of conceptual variability across speakers but postulate nonetheless that the notion of similarity should pertain to a more stable or invariant part of the conceptual structure. Prototypes (Rosch, 1973; Rosch, 1975; Rosch, 1978) could form such a proposal for conceptual invariance, and yet they also prove sensitive to context (Roth and Shoben, 1983). Moreover, the stability of prototypical structure across subjects may not be as high as originally demonstrated, as Barsalou (1987) showed on a large-scale replication study that inter-subject agreements on prototypes ranged between 0.45 and 0.50, significantly below the original 0.90 reported by Rosch (1975).

Assessing conceptual similarity experimentally is subject to many interfering parameters. One of them, as we previously mentioned, is *knowledge* (Goldstone and Son, 2012). Several proposals have been made to bypass knowledge interference, one of them being to experiment on dummy or artificial concepts which specifically require no previous knowledge from tested subjects (Murphy, 2002, p. 141). Yet again, similarity judgments based on artifact categories have proven unreliable as artifact categories are unstable and depend on the categorization task at hand (Sloman and Malt, 2003; Malt and Sloman, 2007).

In short, conceptual similarity remains *hard* to validate experimentally, and is more often than desired a matter of seeing the glass as half full: speakers never significantly disagree on their similarity judgments, but they never totally agree either. The pervasiveness of conceptual variability has gradually worked its way through cognitive science, and much recent work now take for granted that conceptual representations can never be assumed to be fully identical across speakers, given that they are essentially grounded in different background experiences (e.g., Connell and Lynott, 2014, p. 400). For some, it should be relatively easy to come to term with the idea that speakers hold rather different concepts, given how often linguistic communication actually requires clarification (Yee and Thompson-Schill, 2016, p. 1024). For many, however, this still does not necessarily represent an obstacle to successful communication, as what matters ultimately is that speakers are able to coordinate *during* conversation to align their conceptual representations on aspects relevant to the situation under discussion (e.g., Pickering and Garrod, 2006; Connell and Lynott, 2014). We now turn to a historical overview of those approaches and to what their formal characterizations entail.

### 2.3 From Coordination to Alignment

As we have previously detailed in Section 2.1, linguistic communication requires *cooperation* and *coordination* between interlocutors in that it notably involves speakers doing things with words while trying to have their addressees recognize their intentions (Clark, 1992, p. xii). As Clark (1996) emphasized, there is more to language than just a speaker speaking and a listener listening, thus linguistic communication cannot be reduced to mere signal processing. Several research have therefore since proposed to approach (linguistic) communication as *alignment of information states* rather than *information transfer* (e.g., Pickering and Garrod, 2004; Pickering and Garrod, 2006; Garrod and Pickering, 2009; Pickering and Garrod, 2013; Wachsmuth et al., 2013). Speakers and addresses, they argue, are not rigid entities but interactive agents, constantly negotiating meaning during conversation while relying on dynamic and perpetually evolving conceptual representations. Coordination, then, should be understood as the process by which interlocutors converge to similar if not identical mental representations during conversation, a process referred to as *alignment* (Pickering and Garrod, 2004, p. 172).

Interactive-alignment-based models of linguistic communication such as (Pickering and Garrod, 2004; Pickering and Garrod, 2006) distinguish what they call *situation models* from *linguistic representations* and *general knowledge*. A situation model is defined as a multi-dimensional representation of the situation under discussion—encoding space, time, causality, intentionality and reference to main individuals under discussion (Zwaan and Radvansky, 1998)—and is assumed to capture what people are “thinking about” during conversation. The embodied (and embedded) approach to cognitive science operates a similar distinction between *representations* and *concepts*. A *representation* refers to a “specific, situated, contextual instantiation of one or more concepts necessary for the current task”, while a concept refers to “a general, aggregated, canonical (i.e., contextfree) aspect of experience that has the potential to form the basis of an offline representation” (Connell and Lynott, 2014, pp. 391–392). The distinction between (online) representations and (offline) concepts allows the aformentioned approaches to overcome the challenge posed by conceptual variability to communication: offline concepts may differ widely across interlocutors, successful communication remains possible provided that online representations—or situation models—can be aligned (see, e.g., (Pickering and Garrod, 2006, p. 204) or (Connell and Lynott, 2014, p. 400)).

The way in which those approaches accommodate conceptual variability remains nonetheless quite relative, all things considered. First of all, because they assume coordination to play a key role in the socialization hypothesis itself. Indeed, they do not expect concepts and representations to develop in isolation, but rather to mutually influence one another: online representations or situation models are expected to draw upon both linguistic and general (conceptual) knowledge (Connell and Lynott, 2014, pp. 391–392) while, in return, *online perception affects offline representation* (see Principle 1 in Connell and Lynott, 2014, p. 393). Moreover, they assume that alignment at one level of representation will enable or improve alignment at other levels (Pickering and Garrod, 2004, p. 172) so that speakers are expected to align their general knowledge—and the underlying concepts—alongside their situation models throughout coordination (Pickering and Garrod, 2006, p. 215). Consequently, coordination is considered to act as a catalyzer of conceptual similarity: it is not only that speakers of the same community will be better able to coordinate thanks to the similarity of their conceptual spaces—itself deriving from the similarity of their background experiences—it is also that repeated coordination between them will in turn increase their overall conceptual similarity, ultimately leading to a virtuous circle of mutual understanding across speakers of the same community.^2^

Second of all, and more importantly, the tolerance of the aforementioned approaches to conceptual variability remains all relative in that they still consider similarity between background experiences to constitute a prerequisite to successful alignment, coordination and therefore communication. As Garrod and Pickering (2009) point out, “alignment is typically achieved […] because people start off at a very good point. They communicate with other people who are largely similar to themselves, both because they process language in similar ways and because they share much relevant background knowledge” (see p. 294). As such, they rest upon a strong interpretation of the socialization hypothesis, where it should *not* be possible for any two speakers to coordinate and therefore successfully communicate if their respective conceptual spaces remain grounded in fundamentally different background experiences. In fact, the socialization hypothesis still remains a prerequisite to successful communication.

Those considerations invariably lead us to question how strictly we should understand the notion of alignment so far defined to entail *identicity* of conceptual representations. After all, given that online representations are expected to draw upon both linguistic and offline conceptual knowledge, alignment should always be partial at best (Pickering and Garrod, 2006, p. 215). But the interactive-alignment-based models remain heavily grounded in the Shannon–Weaver code model of communication (Shannon and Weaver, 1949) and as such they still often explicitely consider *identicity of messages* between interlocutors to define communication success (see, e.g., Pickering and Garrod, 2013, p. 329). Yet again, this identicity constraint is often relaxed to mere similarity or sufficient overlap (e.g., Connell and Lynott, 2014, p. 400) and successful communication under conceptual misalignment is then considered possible, but only in as much as misalignment pertains to aspects of conceptual knowledge that are irrelevant to the conversation at hand (Pickering and Garrod, 2006, p. 215). The following example, adapted from (Connell and Lynott, 2014, p. 401) illustrates how, in fact, alignment may not always equate *agreement* but sometimes mere *compatibility* between conceptual representations:

[…] imagine your lifetime experience of dogs has been entirely of the small, handbag-dog variety, and that you are unaware that dogs come in any form larger than a chihuahua. You then meet someone who has only ever experienced large working dogs and is unaware that dogs come in any form smaller than a German shepherd. An exchange such as “Do you like dogs?” “Yes, we have one at home,” “Same here, we just got one last week from the shelter,” is perfectly effective communication where each party understands the other, even though each individual is representing quite a different dog in both canonical (i.e., liking dogs in general) and specific (i.e., my pet dog at home) forms […]

The question, then, pertains to the prevalence of compatibility: should it be considered the norm rather than the exception? And how far does it extend? For if indeed the notion of similarity so far considered actually tolerates extreme ranges of variability and negligible overlap between conceptual representations, then it becomes rather devoid of content. Even more so if, as we later show in Section 6, compatibility emerges from idiosyncrasies in speakers’ background experiences, so that alignment can be satisfied even with conceptual representations grounded in fundamentally different background experiences. And the socialization hypothesis then becomes unnecessary, if not inoperative. Before we turn to a more formal investigation of the questions at hand, let us detail several remaining theoretical and methodological challenges.

### 2.4 Remaining Obstacles to the Formal Characterization of the Socialization Hypothesis

As we have previously emphasized in Section 2.1, the socialization hypothesis is first and foremost a hypothesis about conceptual *spaces*. As such, it rests upon a very important property of human cognition at large, namely, that the conceptual space *has structure* (Gärdenfors, 2004; Gärdenfors, 2014).

This particular emphasis on the structure of the conceptual space stresses the need to operate a distinction between latent *structure* and *surface* form, especially when it comes to alignment. This distinction is all the more important that Wachsmuth et al. (2013) underlined that the two do not necessarily go hand-in-hand, for, first, *superficial alignment does not necessarily guarantee structural alignment* (see p. 5). In the particular case of conceptual similarity that concerns us here, this notion of *surface form* can be understood as the behavioral response subjects typically exhibit on various cognitive tasks—such as lexical similarity judgments—the only type of empirical evidence actually accessible to us in practice, for conceptual representations within subjectivist or cognitivist approaches remain mere theoretical constructs. Yet, the problem is, as it has been long argued, that behavioral correlates between subjects on such tasks do not guarantee identicity of concepts (see, e.g., (Davidson, 1984, p. 163), or (Pelletier, 2017, p. 52)). Indeed, Gentner (1988), for instance, showed that adults and children below 8 years old respond differently to the question “how is a cloud like a sponge?”: children, unlike adults, are more inclined to favor the attributional interpretation that “they are both soft and fluffy” over the relational one that “they can both hold water and give it off later”. Such differences in response patterns typically exemplify discrepancies across subjects’ underlying concepts of Cloud and Sponge, and across their relationships to other concepts such as Water or even Fluffiness. Those apparent discrepancies, however, do not preclude mutual agreement on their respective judgments of similarity with respect to Cloud and Sponge.^3^

Conversely, Wachsmuth et al. (2013) argued that *superficial variability does not necessarily imply structural misalignment* (*ibid.*). Here again, one must bear in mind that the socialization hypothesis pertains to a *whole* that is more than just the *sum of its parts*. Yet, due to the practical limitations of experimenting on human subjects, the type of conceptual variability reported in Section 2.2 is almost systematically aggregated on a (very) limited set of entries that may not be representative of the conceptual space *as a whole*. Therefore, it is perfectly possible that such empirical evidence does not actually call into question the socialization hypothesis, for it may not actually prevent a characterization of *overall* similarity between conceptual spaces. Even more so if we are to take into account the *division of linguistic labor* previously detailed in Section 2.2, which suggests that variations across speakers’ conceptual representations may be unevenly distributed across the entire conceptual space, and that high local variability is actually to be expected. Thus, in addition to developing experimental protocols that allow for testing conceptual similarity across the *entirety* of the conceptual space, it appears necessary to develop measures of conceptual similarity that quantify the overall structural similarity between any two spaces, while potentially tolerating high degrees of local and superficial variability.

To overcome parts of the aforementioned challenges, we propose to resort to distributional semantic models of lexical meaning. Indeed, we argue that those models prove particularly suited for the modeling task at hand, given that 1) they provide full control over speakers’ background experiences; 2) their geometric structure allows for defining two distinct notions of similarity: a) at the superficial level, between any two elements, through the notion of cosine *similarity* which models humans behavioral response to lexical similarity tasks; and b) at the structural level, between any two distributional models, through the notion of transformational alignment which makes it possible to quantify similarity over entire spaces, rather than a set of limited entries; and 3) their overall generation pipeline parallels that of human processing and conceptual formation in a cognitively plausible way. We now turn to their formal introduction.

## 3 Distributional Semantic Models

### 3.1 Definition

Distributional Semantic Models (DSMs; Turney and Pantel, 2010; Clark, 2012; Erk, 2012; Lenci, 2018) can be formalized as tuples <T,C,F,S>, meaning that a set of targets *T* is represented in terms of a function *F* of the frequency of co-occurrence of its elements with a set of contexts *C*. *S* is then a measure defined over T×T that yields results interpreted as similarity judgments. DSMs have been shown to successfully account for a number of linguistic phenomena, both at the word and sentence level (see Lenci, 2018, for an overview). Their success, however, is dependent on the exact shape of the model, in particular its architecture and hyperparameters, and the fine-tuning of each of the components has been widely explored in the literature (Bullinaria and Levy, 2007; Bullinaria and Levy, 2012; Baroni et al., 2014; Kiela and Clark, 2014; Lapesa and Evert, 2014; Levy et al., 2015).

DSMs come in two notable variants: *count-based* models such as those originally used for Latent Semantic Analysis (Landauer and Dumais, 1997) and *prediction-based* models which create dense representations for words by learning to predict target words and/or context words using neural networks (e.g., Collobert and Weston, 2008; Mikolov et al., 2013a; Mikolov et al., 2013c). Although Baroni et al. (2014) originally argued that prediction-based DSMs outperform their count-based counterparts, Levy et al. (2015) and Mandera et al. (2017) have since shown that both count and predict models could perform equally well provided specific modeling adjustments and hyperparameters tuning, especially as Levy and Goldberg (2014) showed that certain implementations of prediction-based models are actually equivalent to count-based ones in that they actually perform implicit matrix factorization of the PMI weighed word-context matrix. Despite all considerations, count-based models remain the more direct implementation of the distributional hypothesis of Harris (1954) and are still considered solid options for meaning representation, especially because of the increasing necessity to have transparent and explainable models.

In a traditional count-based model distributional representations of words are computed by aggregating co-occurrence counts of context words found on both sides of a target within a specified range called the *window size*. A given entry of the raw count matrix, corresponding to the row index of a target word *w* and the column index of a context word *c* is then weighted using Positive Pointwise Mutual Information (PPMI):

PPMI=max(PMI(w,c), 0) (1)

where the PMI for *w* and *c* is given by:

$$\text{PMI}\left(w,c\right)=\log\frac{P\left(w,c\right)}{P\left(w\right)\cdot P\left(c\right)}$$ (2)

In order to reduce the dimensionality of the T×C matrix and to capture higher order co-occurrences that are latent in the data, the sparse PPMI matrix of word vector representations *W* is then converted to a dense matrix using Singular Value Decomposition ~ (SVD):

$$W=U\cdot \text{Σ}\cdot V^{⊤}$$ (3)

where *U* is the matrix of (left) singular vectors, Σ is the matrix of singular values, and *V* is the matrix of (right) singular vectors. *W* is then reduced to a low-dimensional matrix $W_{d}$ by selecting the top *d* singular vectors ranked in decreasing order of singular values:

$$W_{d}=U_{d}\cdot {\text{Σ}}_{d}^{\alpha }$$ (4)

where the exponent α∈[0,1] is a hyperparameter which has been shown to positively impact performances on some specific semantic tasks (Caron, 2001; Bullinaria and Levy, 2007; Bullinaria and Levy, 2012; Levy et al., 2015).^4^

The usual motivation behind dimensionality reduction is to drop factors that account for little variability in the original weighted PPMI matrix. In the particular case of SVD described above, the reduced matrix $W_{d}$ is often referred to as the *best rank-d approximation* (e.g., Martin and Berry, 2007, p. 41). The choice of the first *d* dimensions therefore relies on a variance-preserving assumption: as the obtained $W_{d}$ matrix is the one that best approximates, among matrices of rank *d*, the original PPMI matrix, it should also be the one that better represents the desired semantic space. Yet, while the hyperparameters’ space has been widely explored in the literature, this assumption has hardly ever been questioned. Interestingly, we show in the following section that the preservation of the total variance in the original matrix is marginal at best, casting doubts on the original motivation behind this variance-preservation bias. As we will later show in Section 4.1, calling into question the variance-preservation bias proves determinant in investigating the socialization hypothesis, in that it concretely allows us to model coordination and conceptual alignment within the distributional semantics framework with only marginal modifications to the traditional DSM generation pipeline. Indeed, we show in Section 5.1 that it is actually possible for DSMs to capture different kinds of semantics relations from the same corpus, so that rather than generating a *single* meaning space from the PPMI matrix, a *collection* of possible meaning spaces could coexist within the same set of data. Coordination then becomes the process of *dimensionality sampling*, that is, the process of reducing the SVD matrix by selecting the set of singular vectors that best satisfy the coordination constraints under consideration, rather than those that best preserve the variance.

### 3.2 The Variance-Preservation Bias

Bullinaria and Levy (2012) originally questioned the importance of the top singular vectors in the SVD matrix and suggested removing the first 100 dimensions, claiming that the highest variance components were influenced by aspects that turned out to be irrelevant to lexical semantics. Their observation remained nonetheless largely ignored in the literature, and it is only very recently that research formally questioned the process of dimensionality selection in DSMs (Mu and Viswanath, 2018; Raunak et al., 2019) ultimately bringing further supporting empirical evidence to the original claim of Bullinaria and Levy (2012).

The process of dimensionality selection can be motivated by slightly different considerations: 1) creating compact and computationally efficient vector representations, which can even lead to significant performance improvement (Landauer and Dumais, 1997; Bullinaria and Levy, 2012); 2) reducing some undesirable geometrical effect in the original vector space (Grefenstette, 1994, p. 102); or even 3) mitigating the noise intrinsically present in partial data and increasing the robustness of the model (Deerwester et al., 1990). Regardless of the underpinning motivation, the dimensionality reduction process considered here remains a *lossy* process, where part of the data may be deliberately discarded following specific modeling considerations. In that sense it is to be distinguished from *rebasing* and potentially *lossless* methods which may be able to align the dimensionality of the reduced space to the original data matrix rank. An example of such approaches is *multidimensional scaling* (MDS; Shepard, 1962a; Shepard, 1962b) where similarity ratings on sets of word pairs are first collected among human subjects, before attempting to account for the entirety of the collected data via a few potentially meaningful latent dimensions in order to further explore the notion of similarity under study (Heider and Olivier, 1972; Ross and Murphy, 1999).^5^

The *best rank-d SVD approximation* that interests us here, however, is historically grounded in methodological considerations coming from image processing and more specifically image compression (e.g., Andrews and Patterson, 1976a; Andrews and Patterson, 1976b). Given an image represented as a matrix of pixels, the frequent correlation between nearby pixels in images will allow for the creation of low-dimension representations with only a few singular vectors accounting for most of the variance in the original data (Strang, 2016, p. 365). Variance-preservation is quantified via the notion of matrix *energy* (*E*), formally defined as the square of the Frobenius norm of the data matrix and also equal to the sum of the squared singular values of the data matrix SVD (see Eq. 5).

$$E_{W}=||W|{|}_{F}^{2}=\overset{n}{\underset{i=1}{\sum }}\overset{m}{\underset{j=1}{\sum }}|w_{i,j}{|}^{2}=\overset{\text{min}\left\{m,n\right\}}{\underset{i=1}{\sum }}\lambda_{i}^{2},with\{\begin{array}{l} W\in {\mathbb{R}}^{m\times n}\\ W=U\cdot \left[\begin{array}{ccc} \lambda_{1} & & \\ & \ddots & \\ & & \lambda_{\text{min}\left\{m,n\right\}} \end{array}\right]\cdot V^{⊤} \end{array}$$ (5)

A traditional *rule of thumb* for SVD dimensionality selection in image processing is to try and retain about 90% of the original energy (Leskovec et al., 2014, p. 424). Yet, as we can see in Table 1, this is far from being the case when selecting the top 300 dimensions of the SVD on a standard PPMI-weighted count-based DSM model, as the preserved energy remains systematically below ∼15%. Moreover, results on *d* = 10,000 suggest that the aforementioned rule of thumb is difficult to apply as-is to DSMs as it leads to high-dimensional and therefore computationally inefficient models.

**TABLE 1:** Percentage of total energy preserved with d=10 000 and d=300 top dimensions for DSMs trained on various corpora described in Table 2.

	d=10 000	d=300
WIKI07	66%	11%
OANC	72%	11%
WIKI2	58%	10%
ACL	62%	13%
WIKI4	52%	9%
BNC	59%	10%
WIKI	39%	9%

All models are PPMI-weighted count-based DSMs generated with a window of 2.

**TABLE 2:** Corpora used to generate DSMs

Corpus	Word count	Details
OANC	17M	Open american national Corpus.^a^ includes both spoken and written language, ranging from telephone and face-to-face conversations to letters, fiction, technical reports, newspapers or travel guides
WIKI07	19M	0.7% of the English wikipedia (WIKI) sampled across the entire dump
ACL	58M	Association for computational linguistics (ACL) anthology References corpus (Bird et al., 2008). Contains research papers in computational linguistics exclusively
WIKI2	53M	2% of the English wikipedia (WIKI) sampled across the entire dump. WIKI2 contains 12.5% of WIKI07
BNC	113M	British national Corpus.^b^ includes both spoken and written language, ranging from informal conversations and radio shows to newspapers, academic books, letters or fiction
WIKI4	106M	4% of the English wikipedia (WIKI) sampled across the entire dump. WIKI4 contains 15% of WIKI07 and 100% of WIKI2
WIKI	2 600M	Full English wikipedia dump of January 20, 2019, generated and preprocessed (tokenize and lowercased) with WiToKit^c^ based on wikiextractor^d^ and polyglot (Al-Rfou et al., 2013). WIKI contains 100% of WIKI07, WIKI2 and WIKI4

a https://www.anc.org/OANC/index.html

b http://www.natcorp.ox.ac.uk/

c https://github.com/akb89/witokit

d https://github.com/attardi/wikiextractor

**Algorithm 1:** Absolute orientation with scaling AOS(A, B)

Compute the sum of outer products $H={\sum^{\text{​}}}_{i=1}^{n}b_{i}^{T}a_{i}$
Decompose $\left[U,S,V^{T}\right]=\text{svd}\left(H\right)$
Build rotation $R=UV^{T}$
Rotate $\tilde{B}=BR$ so each ${\tilde{b}}_{i}=b_{i}R$
Compute scaling $s={\sum^{\text{​}}}_{i=1}^{n}\langle a_{i},\tilde{b_{i}}\rangle /||\tilde{B}|{|}_{F}^{2}$
**return** $\overset{˘}{B}$ as $\overset{˘}{B}\leftarrow s\tilde{B}$ so for each $\overset{˘}{b_{i}}=s\tilde{b_{i}}$

**FIGURE 1:**
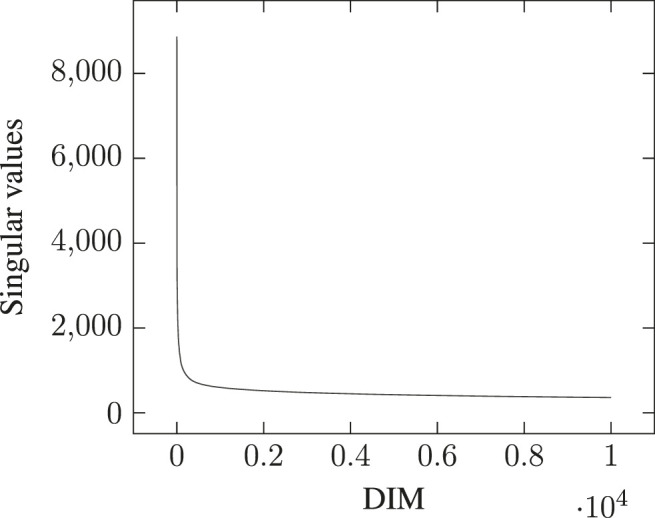
Distribution of singular values across [0, 10,000] top dimensions for a PPMI-weighted count-based DSM generated on the full English Wikipedia (WIKI) corpus detailed in Table 2, with a window of 2.

This issue, however, is barely mentioned in the literature: Bullinaria and Levy (2007) explain that dimensionality reduction is performed with minimal loss defined using the standard Frobenius norm, but do not quantify it (see p. 897). Earlier work using SVD for Latent Semantic Analysis state that many of the latent singular values remain small and can therefore be ignored (Deerwester et al., 1990, p. 395). But this observation is misleading: as we can see in Figure 1, the distribution of singular values follows a highly-skewed Zipfian curve, so that the latent components may indeed quickly appear very small in comparison to the top components. However, the tail of the distribution remains quite *long*, especially as Table 1 suggests the matrix rank to be significantly higher than 10,000. The cumulative effect of the tail’s length can therefore be so that retaining only a few top components, even if those correspond to significantly higher singular values, may prove to account for only a tiny portion of the total energy. Be that as it may, the most frequent observation supporting the choice of a limited number of top components in the SVD remains that models simply “work” as-is, and the double benefit of having both computationally efficient and effective models frees authors from having to investigate further the consistency of their modeling choices (e.g., Lund and Burgess, 1996).

### 3.3 Cognitive Plausibility of DSMs

Determining whether DSMs constitute cognitively plausible models first requires asking what DSMs are supposed to be models *of*. And yet the answer to that question appears to be far from consensual: Sahlgren (2008), for instance, insists that distributional models are models of word meaning “as they are in the text” and not “in the head”, so that DSMs should be considered primarily as computational models of meaning rather than “psychologically realistic model[s] of human semantic processing” (Sahlgren, 2008, pp. 134–135). Meanwhile, Günther et al. (2019) consider that DSMs stand in the long tradition of learning theories which postulate that humans are excellent at capturing statistical regularities in their environments. Yet, even if we are to agree with Günther et al. (2019), we must acknowledge that Sahlgren (2008) raises an important question: can distributional information found in corpora be considered representative of the type of distributional information grounding humans’ conceptual representations in the first place?

#### 3.3.1 DSMs Are Not Grounded in Sensorimotor Experience

The first challenge faced by DSMs in their lack of *grounding* in sensorimotor experience of the real world, which makes them theoretically problematic as a sole account of meaning (e.g., De Vega et al., 2008; Wingfield and Connell, 2019). And indeed, Landauer and Dumais (1997) originally acknowledged that “to be more than an abstract system like mathematics, words must touch reality at least occasionally” (see p. 227). The problem is probably best illustrated by Harnad (1990) and his *Chinese/Chinese dictionary-go-round* example, itself an extension Searle’s *Chinese Room argument* (Searle, 1980): if one only had access to a Chinese/Chinese dictionary in order to learn the Chinese language, one would soon find themselves locked into a symbol/symbol merry-go-round that would render the task impossible (Harnad, 1990, pp. 339–340). As Glenberg and Mehta (2008) further note, no amount of statistical information can actually solve the problem of the circularity of definitions, if one cannot resort to alternative grounded modalities to understand what words actually *mean* (see p. 246).

By and large, such considerations raise the question of whether the type of *linguistic* distributional information found in text can be reasonably assumed to adequately mirror more *general* distributional information found in the world. As Connell (2019) puts it:

Linguistic distributional statistics and simulated distributional statistics contain similar patterns, but do not directly reflect one another. In contrast to linguistic information, which comprises statistical regularities between word forms, simulated information encodes statistical regularities at the level of meaning due to the inclusion of situational context in simulated representations. A car, for instance, typically has wheels and a driver, operates on the road or street, and sometimes needs a service or repair. Objects, events, and other situational entities tend to occur together in the real world in ways that, through cumulative interactive experience, can give rise to statistical patterns of how referent concepts are distributed in relation to one another.

This question then extends to the question of the representativeness of *linguistic* distributional information in and of itself, and to whether what is found in standard DSM training corpora can be considered—both *quantitatively* and *qualitatively*—to constitute a representative sample of the type of linguistic distributional information humans are exposed to (Wingfield and Connell, 2019, pp. 8–11).

Yet, despite Connell’s concerns, several investigations have actually considered language to mirror the real world in ways that distributional information found in text could be assumed to reflect, in part or in full, distributional information grounded in sensorimotor experience (see, e.g., Barsalou et al., 2008; Louwerse, 2011). Be that as it may, what is important for our purpose here is not that distributional patterns found in corpora constitute *comprehensive* samples of distributional information grounding humans' conceptual representations, but only that they condition the structural properties of the conceptual space in a plausible fashion (see more details in Section 3.3.2). Furthermore, insofar as the distributional hypothesis remains a hypothesis about *cascading variations*—*more* similar background experiences should entail *more* similar conceptual spaces—emphasis should be put on modeling plausible *differences* across distributional patterns speakers may be exposed to. We will return to that question in greater length in Section 6.

#### 3.3.2 Can DSMs Nonetheless Model Conceptual Knowledge?

DSMs have historically been considered to model *conceptual* aspects of meaning, given how successful they prove to be at performing conceptual tasks such as *lexical similarity*, *priming* or *analogy* (see Westera and Boleda, 2019, §3.2). But can the vector for “cat” in a standard DSM really be considered to model the concept Cat when indeed it is only an abstraction over occurrences of the *word* cat and not over occurrences of *actual* cats? For Westera and Boleda (2019) it should not, and DSMs can at best be claimed to model *concepts of words* but definitely *not* concepts. And this distinction has its importance as, for them, one cannot expect relations that hold between concepts to necessarily hold between concepts of words. For example, the entailment relationship that may exist between Cat and Animal may not necessarily hold between TheWordCat and TheWordAnimal.

Insofar as those considerations derive from the lack of grounding of DSMs previously detailed in Section 3.3.1, we will argue along the same lines. That is, we will not argue that DSMs provide comprehensive models of the conceptual space as a whole, but only that they provide satisfactory approximations for the purpose at hand. Our emphasis throughout this work being on the *structure* of the conceptual space—especially with respect to alignment—rather than, say, its cardinality, we remain mainly interested in the distribution of information across the dimensions of the DSM, and how that might be able to capture and reflect some structural properties of the conceptual space. In response to Westera and Boleda (2019), we will therefore say that, after all, concepts of words *are* concepts, so that even though DSMs were only able to model concepts of words, they could still be characterized as *subspaces* of a larger conceptual space, governed by similar constraints and structural properties: what matters here is not necessarily that, e.g., similar entailment relationships that hold between concepts also hold between concepts of words, but that *a* notion of entailment could be characterized in both the space of concepts and the subspace of concepts of words.

#### 3.3.3 When DSMs Parallel Human Cognition

As we have previously mentioned, DSMs stand in the long tradition of learning theories which postulate that humans are excellent at capturing statistical regularities in their environments (Günther et al., 2019, p. 6). And in fact, as Connell and Lynott (2014) note, “natural languages are full of statistical regularities: words and phrases tend to occur repeatedly in similar contexts, just as their referents tend to occur repeatedly in similar situations” (see p. 395). Humans, as it appears, are sensitive to those regularities (e.g., Aslin et al., 1998; Solomon and Barsalou, 2004; Louwerse and Connell, 2011) which allows them to build conceptual representations from distributional knowledge (e.g., McDonald and Ramscar, 2001). Children, for example, are known to exploit statistical regularities in their linguistic environments, either via simple conditional probabilities when segmenting speech streams into words (Saffran et al., 1996), or via distributional patterns when acquiring syntactic knowledge (Redington et al., 1998).^6^

Jenkins (1954) originally proposed a summary of the whole lexical acquisition process: “intraverbal connections arise in the same manner in which any skill sequence arises, through repetition, contiguity, differential reinforcement” (see p. 112). Since then, several research have argued that the learning of associations between stimuli is driven by *contingency* rather than *contiguity* (Rescorla and Wagner, 1972).^7^ As Rescorla (1968) details, the notion of contingency differs from contiguity in that it takes into account not only what *is* there but also what *is not* in the form of conditional probabilities. In essence, the notion of contingency characterizes the *informativity* of a given stimuli. For Günther et al. (2019), PPMI-based DSMs directly follow such learning theories as they indeed encode *mutual information* between words and contexts, that is, their respective informativity, rather than raw word-context co-occurrence count.

A crucial aspect of DSMs is that they follow the emergentist approach to cognitive development (e.g., Elman et al., 1996) and conceptual representations (e.g., Rogers and McClelland, 2004) in considering that long-term knowledge is an emergent representation abstracted across multiple experiences. Within the emergentist family of connectionist models, there is no real distinction between knowledge of something and knowledge of the contexts in which that thing occurs, and several implementations have historically been proposed to show how a conceptual representation could be abstracted from contextual experience (e.g., Elman, 1990; Elman, 1993; Altmann, 1997).

For Jones et al. (2015) both the connectionist and the distributional approaches have in common to hypothesize the existence of a *data reduction* mechanism that enables focusing on important statistical factors that are constant across contexts while throwing away factors that are idiosyncratic to specific contexts (see p. 240). Landauer and Dumais (1997) argued early on that the dimensionality reduction step in the DSM generation pipeline could model the transition from episodic to semantic memory,^8^ formalized as the generalization of observed concrete instances of word-context co-occurrences to higher-order representations potentially capturing more fundamental and conceptual relations (see p. 217). The idea that DSMs could provide computational models of semantic memory can also be found in (McRae and Jones, 2013; Jones et al., 2015).

Another important assumption made with respect to this compression mechanism is that it relies on a form of covariation-based decomposition of the previously aggregated stimuli. As such, it operates in a similar fashion than Singular Value Decomposition (SVD) or Principal Component Analysis (PCA) in being able to structure and organize latent information based on variance: broad, high-order distinctions come first before more fine-grained ones (Rogers and McClelland, 2004; Jones et al., 2015). This assumption is supported by empirical evidence showing that children acquire concepts through progressive differentiations: 18-months-olds first develop global conceptual categories such as *animals*, *vehicles*, *plants*, *furniture* and *kitchen utensils* before being able to operate high-constrat basic-level distinctions among those categories by 30 months, and ultimately learning to operate low and moderate basic-level contrasts among those categories later one (Mandler et al., 1991).

Now Glenberg and Mehta (2008) argued that covariation among words is not sufficient to characterize meaning, and showed that participants failed to rely on covariance structure to, e.g., classify unnamed features for familiar domains. Yet, this does not mean that covariation cannot be used as a proxy to capture certain conceptual properties such as lexical similarity. Again, the fact that concepts cannot be characterized by covariation alone does not make it useless. Note again here, as Landauer and Dumais (1997) have stressed before, that we do not need to consider SVD to constitute *the* cognitive mechanism used by humans to perform data compression. We can just assume that the brain uses some sort of dimensionality reduction mechanism *akin* to SVD in order to create abstract conceptual representations by favoring high covariance structure while eliminating idiosyncrasies.

**FIGURE 2:**
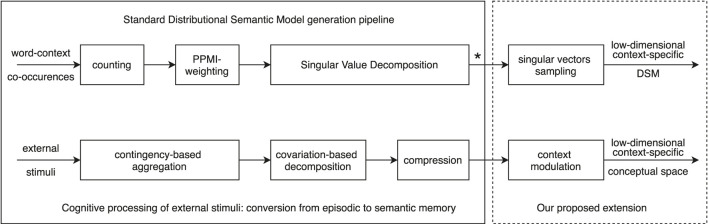
Parallel between the standard DSM generation pipeline (upper part) and human cognition (lower part). The left part of the diagram details the cognitive processing of external stimuli converting episodic memory to semantic memory, while the right part is our proposed extension for modeling ad-hoc coordination (detailed in Section 4.1). Note that the standard output of a traditional DSM generation pipeline (see *) is a low-dimensional matrix of dimension k≈300 made of top variance-preserving components. As we detail in Section 4.1, we replace this by top k=10 000 components from which we sample a subset of singular vectors.

In short, the standard DSM generation pipeline can be considered to parallel human cognition via three specific processes (see Figure 2): 1) *contingency-based aggregation* of distributional information through word-context co-occurence counting and PPMI-weighting; 2) covariation-based *decomposition* through Singular Value Decomposition; and 3) *compression* through dimensionality reduction of the SVD matrix.

## 4 Model and Experimental Setup

### 4.1 Modeling Coordination as Singular Vectors Sampling

Recall the “dog” example of Connell and Lynott (2014) previously introduced in Section 2.3: imagine yourself discussing *dogs* with someone who has only ever encountered dogs the size of a German shepherd while you have only encountered dogs the size of a chihuahua. At the beginning of the conversation, those differences across background experiences could translate as differences across your respective similarity judgments: assuming here for the sake of the argument that all similarity judgments are solely based on a *size* feature, you may think that Dog is more similar to Cat or even to Mouse than to Bear, while your interlocutor may think the opposite. Yet, provided that you talk long enough, you and your interlocutor may somehow accommodate those discrepancies across your respective background experiences and update your conceptual representations of *dogs* accordingly. This may in turn translate as cascading updates in your similarity judgments, and at the end of the conversation you may then both consider Dog to be more similar to Cat than to Bear, and to be more similar to Bear than to Mouse.^9^

In this work we propose to characterize superficial alignment during ad-hoc coordination as the cooperative act of *aligning lexical similarity judgments* on a limited set of word pairs. In practice, we propose to model coordination with DSMs as *singular vectors sampling*: we modify the standard DSM generation pipeline by replacing the ill-motivated variance-preservation bias described in Section 3.2 by an explicit coordination bias, sampling the set of *d* singular vectors which maximize the correlation with a particular lexical similarity dataset. The core assumption underlying our sampling algorithm is that it is actually possible for DSMs to capture different kinds of semantic relations from the same corpus, so that rather than generating a *single* meaning space from the PPMI matrix, a *collection* of possible meaning spaces could coexist within the same set of data. The collocates of *cat*, for instance, could provide enough information to characterize it as similar to *tiger* on the one hand (i.e., having a neighborhood of ontologically-related words), or to *meow* on the other hand (i.e., having a neighborhood of generically related words), and be aggregated in different dimensions during the factorization step. This assumption will be supported later on by our experimental results showing that DSMs relying on our sampling algorithm rather than the variance-preservation bias can indeed perform significantly better on several lexical similarity datasets, as different dimensions encode different semantic phenomena (see Section 5.1).

In practice, given that the rank *r* of the sparse PPMI matrix is usually well beyond a manageable order of magnitude (*r* > 100,000) to explore all possible subsets in *U*, we propose a sampling algorithm to efficiently sample only a limited number of subsets of singular vectors in *U*. Our sequential (seq) sampling algorithm works in two passes:

1. add: during the first pass, the algorithm iterates over all singular vectors and selects only those that increase performance on a given dataset;
2. reduce: during the second pass, the algorithm iterates over the set of added singular vectors and removes all those that do not negatively alter performance on the given dataset.

The structure of the algorithm, especially the presence of the reduce step, is motivated by the presence of many complex semantic redundancies across singular vectors from the point of view of fitting a particular meaning space, so that adding a particular singular vector to a set pre-existing ones may make some of them redundant.

Additionally, and for computational efficiency, we reduce the number of singular vectors under consideration by sampling over the top-*k* singular vectors only, with *k* = 10,000.^10^ The algorithm can be run through multiple iterations, and may iterate over singular vectors in linear or shuffled order (of singular value). We apply 5-fold validation and report scores averaged across test folds, with the corresponding standard error. We define performance on a given similarity dataset as both the Spearman correlation *and* the Root Mean Square Error (see Eq. 6) computed on a set of word pair similarities. That is, the sampled models have to align *both* the ranking *and* the absolute similarity values of the set of word pairs with that of the dataset. This feature modeling choice is motivated by preliminary results on k-fold validation showing a tendency to overfit when performance metric is restricted solely to Spearman correlation.^11^

In effect, our model approximates coordination as context modulation, where context modulation is understood as the act of accommodating past experienced contexts to the specific context of the discussion. Indeed, several research have shown that dimensions in DSMs capture different contexts in which words are used (e.g., Griffiths et al., 2007, p. 221) so that, in fact, the process of singular vectors sampling is tantamount to context selection and aggregation. The main benefit of our approach is that it allows us to model cascading conceptual modulation across the *entire* conceptual space. Since latent singular vectors condition the content of *all* semantic representations, sampling a set of singular vectors will not just impact the representations of the lexical items being aligned, but actually the entire conceptual space. Moreover, this mechanism of singular vectors sampling is theoretically very convenient as it relieves us from having to formulate explicit assumptions regarding the latent structure of the conceptual space: cascading modulation will always be conditioned on latent interdependencies which are grounded in shared contextual aggregates across semantic representations.

Note, however, that we do not model conceptual update, neither *during* nor *after* coordination. As a matter of fact, since we assimilate coordination to the act of *accommodating existing knowledge* to the situation at hand, we do not actually need to update the original PPMI matrix, which relieves us from having to formulate a theory about how conceptual update could and should proceed in such situations. Since our main purpose throughout this study is to investigate the dynamics of alignment during ad-hoc coordination, we can actually focus on an approximation of the coordination process between any two arbitrary points in time. Similarly, we do not model online coordination at every step of the process—such as conceptual update occurring at every utterance during real-time communication—as we do not need this level of granularity for the purpose at hand. Once again, this should be seen as an opinionated modeling decision rather than a limitation of our model.^12^

Finally, we exclusively focus here on count-based DSMs given that, as we have seen in Section 3.3 their generation pipeline nicely parallels the functioning of human cognition. Moreover, they provide more transparent, explainable and modular models in comparison to their prediction-based counterparts, which makes it easier to operate cognitively-motivated modeling modifications. It appears difficult indeed to transpose our proposed approach to prediction-based DSMs as-is. The singular vectors sampling mechanism could probably be replaced by a kind of post-processing technique akin to what Mu and Viswanath (2018) have used for instance as a way to somehow bypass the variance-preservation bias. But those postprocessing techniques have yet to be formalized for our purpose and one would loose the benefits of sampling on dimensions that explicitly capture context aggregates. Not to mention additionally that those postprocessing techniques usually rely on linear transformations that are sort of “one-shot” and cannot necessarily easily be made to function incrementally.

### 4.2 Measuring Conceptual Alignment via Matrix Transformation

In the previous section we proposed to characterize superficial alignment during ad-hoc coordination as the cooperative act of aligning lexical similarity judgments on a limited set of word pairs. Recall from Section 2.4, however, that we stressed the need to distinguish superficial from structural alignment when investigating the socialization hypothesis, as the two do not necessarily go hand-in-hand. We argued more specifically in favor of a notion of conceptual similarity that could quantify the overall structural similarity between any two conceptual spaces, while potentially tolerating high degrees of local and superficial variability.

In this section we therefore propose to model structural similarity between two DSMs as the minimized Root Mean Square Error (RMSE; Eq. 6) between them. DSMs are first aligned using *absolute orientation with scaling* (see Algorithm 1 below from Dev et al., 2018, originally Algorithm 2.4 in their paper) where the optimal alignment is obtained by minimizing the sum of squared errors under the Euclidian distance between all pairs of common data points, using linear transformations—rotation and scaling—which do not alter inner cosine similarity metrics and hence preserve measures of pairwise lexical similarity.

The Root Mean Square Error (RMSE) between the two matrices *A* and $\overset{˘}{B}$ is then given by:

$$RMSE\left(A,\overset{˘}{B}\right)=\sqrt{\frac{1}{\left|A\right|}\overset{\left|A\right|}{\underset{i=1}{\sum }}||a_{i}-\overset{˘}{b_{i}}|{|}^{2}}$$ (6)

Note that due to floating point approximations, our computed RMSEs are not symmetric, so that $RMSE\left(A,\overset{˘}{B}\right)\neq RMSE\left(\overset{˘}{A},B\right)$, with $\overset{⌣}{B}=AOS\left(A,B\right)$ and $\overset{⌣}{A}=AOS\left(B,A\right)$. To alleviate this problem, we always report the averaged RMSE: $\bar{\text{RMSE}}=1/2\left[\text{RMSE}\left(A,\overset{˘}{B}\right)+\text{RMSE}\left(\overset{˘}{A},B\right)\right]$.

Our notion of structural similarity follows alignment-based models (Goldstone and Son, 2012, p. 165) in that it attempts to place elements of the two DSM matrices in correspondence with one-another via a set of structure-preserving operations, and therefore does not measure a raw comparison between them. The underlying methodology has been widely used in computational linguistics to align DSMs across languages (e.g., Mikolov et al., 2013b) although it is to be distinguished from other alignment-based approaches in the field which apply potentially non-cosine-preserving linear transformations (e.g., Tan et al., 2015). Such methodologies can also be found in neuroscience with the *hyperalignment* approach put forth by Haxby et al. (2011) which proposes to align patterns of neural response across subjects using linear transformations—namely rotations and reflections—minimizing the Euclidian distance between two sets of paired vectors, in order to abstract away the intrinsic variability of voxel spaces across subjects. The underlying logic is always the same: two models can be transformationally equivalent although they may not appear similar in absolute. Aligning the coordinate system or the basis of two vector spaces, for instance, can uncover measures of relative similarity between two models that otherwise appear radically different when comparing only their original respective coordinate values.

Recall also from Section 4.1 that we proposed to model superficial alignment during coordination with DSMs as singular vectors sampling, with the benefits thereby of being able to model cascading conceptual modulation across the entire conceptual space. The question that arises, then, is if, as defined, superficial alignment will necessarily entail structural alignment. That is, will maximizing the Spearman correlation on a lexical similarity dataset using our singular vectors sampling algorithm on two DSMs generated from two distinct corpora in turn lower the RMSE between them. We report our results on the matter in Section 5.2.

It is important to note here, however, that the connection between our characterizations of superficial and structural alignment are not necessarily obvious. Indeed, our notion of structural similarity satisfies the requirements detailed in Section 2.4 in that it can indeed tolerate high degrees of local and superficial variability: since the RMSE-based structural similarity measures absolute distances between points in space, it is insensitive to relative measures of semantic proximity, unlike what is expected from correlations with lexical similarity datasets. Naturally, if two DSMs have a null RMSE, they will produce identical similarity judgments on a set of word pairs. But the slightest deviation from 0 can have unpredictable consequences depending on the configuration of the space. So in fact, our model makes it possible for any two DSMs to behave very differently with respect to lexical similarity while actually being well aligned structurally (and conversely) following thereby the position of Wachsmuth et al. (2013) detailed in Section 2.4.

### 4.3 Experimental Setup

We generate PPMI-weighted DSMs using a window of size 2 from seven different corpora detailed in Table 2. All corpora are lowercased and tokenized with Polyglot (Al-Rfou et al., 2013). All Wikipedia subsets are generated by sampling the WIKI corpus at the sentence level. Corpora are chosen so as to provide pairs of comparable size (OANC and WIKI07; ACL and WIKI2; BNC and WIKI4) covering different domains and/or different genres (see details in Table 2). Note that our point here, as we have previously detailed in Section 3.3.1, is not to model plausible individual speakers, but plausible *differences* across background experiences. What is important therefore is not that corpora be produced by individual speakers, or even characterize the linguistic experience of individual speakers, but that the differences across their linguistic distributional patterns model plausible differences of background experiences. We return to this question in more details in Section 6. We therefore select corpora which we assume to characterize quite different linguistic distributional patterns: ACL for instance covers exclusively research papers in computational linguistics, while OANC and BNC both include spoken and written language from different genres (newspapers, fiction, technical reports, travel guides, etc.).

For word similarity datasets, we rely on MEN (Bruni et al., 2014), SimLex-999 (hereafter SimLex (Hill et al., 2015); and SimVerb-3500 (hereafter SimVerb; Gerz et al., 2016). MEN is a relatedness dataset containing a list of 3,000 word pairs with a strong bias toward concrete concepts; while SimLex intends to encode *similarity* rather than *relatedness* for 999 word pairs, and provides a more balanced account between *concrete* and *abstract* concepts. Words that have high relatedness in MEN may have low similarity in SimLex. For example, the pair “chicken-rice” has a similarity score of 0.68 in MEN and 0.14 in SimLex. Following previous claims and standard linguistic intuitions, the relatedness dataset MEN should be only weakly compatible with the similarity dataset SimLex: one expresses topical association (i.e. *cat* and *meow* are deemed related) while the other expresses categorical similarity (i.e. *cat* and *dog* might be considered similar in virtue of being members of the same category). Thus, those datasets encode possibly incompatible semantic constraints and it is theoretically impossible to perfectly fit both the meaning spaces they encode with a single DSM. Those two datasets therefore allow exploring our approach across two distinct coordination situations. The third dataset, SimVerb, is a similarity dataset consistent with SimLex, but focusing on verb meaning and providing 3,500 word pairs. Although theoretically compatible with the notion of similarity encoded in SimLex, it focuses on different semantic categories and as such on a potentially different domain with distinct semantic constraints. Given that we rely on MEN and SimLex for pre-validation of our sampling algorithm (see Section 4.1) we add SimVerb as an additional dataset to further check the robustness of our results.

Mincount hyperparameters are set so as to maximize lexical coverage on all similarity datasets while maintaining reasonable overall computing time. We choose a mincount of 2 for OANC, WIKI07, ACL, WIKI2, BNC and WIKI4 and 30 for WIKI. Lexicons are aligned across all DSMs *after* the SVD computation and we obtain a MEN coverage of 93.0% (2,817 pairs out of 3,000), a SimLex coverage of 99.5% (994 pairs out of 999) and a SimVerb coverage of 94.91% (3,322 pairs out of 3,500).

We compute *p* values on each test fold using a Steiger’s test (Steiger, 1980)^13^ following (Rastogi et al., 2015). We consider as the null hypothesis the fact that two models perform identically on a given lexical similarity dataset. We then combine all *p* values for a given k-fold using the weighted harmonic mean (see Wilson, 2019) treating folds as dependent tests, and report a single *p* value per k-fold.

Finally, we make our code available for replication at https://gitlab.com/akb89/avoiding-conflict.

## 5 Results

### 5.1 No Variance-Preservation Bias Means Better Superficial Alignment

We first report the performance of our seq sampling algorithm described in Section 4.1 against PPMI-weighted count-based (TOP) models reduced by selecting the top *n* singular vectors in the SVD matrix, with (α=1) or without (α=0) singular values. In order to provide a completely fair comparison across models, we generate for each fold a specific TOP model with the exact same number of dimensions *n* than the one sampled by our seq algorithm for that particular fold. We similarly compute the statistical significance of the difference of performance between the SEQ and the TOP models *per fold*. We then report a single Spearman correlation per model, corresponding to the *mean* and *standard error* across all 5-folds, and report a single statistical significance score, computed as the harmonic mean of the *p* values across five folds, as previously detailed in Section 4.3.

Our results show that replacing the traditional variance-preservation bias with our sampling algorithm leads to near-systematic improvements on all corpora and across all similarity datasets (see Table 3). The detrimental effect of variance-preservation is first exemplified when comparing DSMs with singular values (α=1) to those without (α=0), an effect originally noted by Caron (2001) and also discussed by Levy et al. (2015). This detrimental effect is then further exemplified by introducing our sampling algorithm and proves most salient on the ACL corpus, with a 17 points increase in performance on MEN, a 13 points increase on SimLex, and a 12 points increase on SimVerb, all statistically significant (p<0.01).

**TABLE 3:** Spearman correlations on MEN, SimLex and SimVerb for DSMs generated from different corpora.

Model	α	WIKI07	OANC	WIKI2	ACL	WIKI4	BNC
		MEN					
TOP	1	0.48 ± 0.01	0.50 ± 0.01	0.53 ± 0.02	0.25 ± 0.03	0.54 ± 0.01	0.61 ± 0.01
TOP	0	0.56 ± 0.01	0.59 ± 0.01	0.61 ± 0.01	0.34 ± 0.02	0.62 ± 0.01	0.69 ± 0.01
SEQ	-	**0.60** ± 0.01	**0.64** ± 0.01	**0.66** ± 0.01	**0.51** ± 0.01	**0.69** ± 0.02	**0.74** ± 0.00
p value		0.0023	0.0003	$<10^{-4}$	$<10^{-4}$	$<10^{-4}$	$<10^{-4}$
ndim		186 ± 5	195 ± 6	200 ± 3	300 ± 9	215 ± 6	161 ± 6
		SimLex					
TOP	1	0.20 ± 0.04	0.18 ± 0.02	0.24 ± 0.02	0.11 ± 0.06	0.25 ± 0.02	0.27 ± 0.03
TOP	0	0.24 ± 0.03	0.22 ± 0.02	0.26 ± 0.02	0.14 ± 0.05	0.27 ± 0.02	0.32 ± 0.03
SEQ	-	0.25 ± 0.03	0.19 ± 0.04	0.30 ± 0.03	**0.27** ± 0.03	**0.38** ± 0.02	**0.41** ± 0.02
p value		0.3802	0.0906	0.0646	0.0001	0.0010	0.0056
ndim		184 ± 12	240 ± 9	196 ± 12	221 ± 5	224 ± 6	201 ± 10
		SimVerb					
TOP	1	0.08 ± 0.02	0.07 ± 0.02	0.11 ± 0.03	0.07 ± 0.01	0.12 ± 0.01	0.16 ± 0.01
TOP	0	0.13 ± 0.01	0.13 ± 0.02	0.15 ± 0.03	0.11 ± 0.01	0.17 ± 0.01	0.22 ± 0.02
SEQ	-	**0.20** ± 0.03	0.19 ± 0.02	**0.21** ± 0.03	**0.23** ± 0.01	**0.25** ± 0.01	**0.29** ± 0.01
p value		0.0019	0.0216	0.0001	0.0015	0.0043	0.0015
ndim		290 ± 17	185 ± 12	317 ± 18	267 ± 13	376 ± 11	331 ± 12

All models are PPMI-weighted count-based models generate with a window size of 2. SEQ models are reduced via our seq algorithm detailed in Section 4.1, while TOP models are reduced by selecting the top n=ndim singular vectors from the SVD matrix, with ndim corresponding for each fold to the number of dimensions sampled by the SEQ model on that fold. All results are averaged across test folds applying 5-fold validation, after taking the best of 10 shuffled runs. Bold results indicate statistically significant differences (p<0.01) between SEQ and TOP (α=0) models.

Explicitly sampling singular vectors leads to an even more interesting observation: *different dimensions encode different semantic phenomena*. Contrary to what was originally argued in (Schütze, 1992, p. 794), all singular vectors are not necessarily meaningful to discriminate particular patterns of word similarities. For example, the semantic phenomenon of *relatedness* encoded in MEN is characterized by a different sampling pattern than the *similarity* phenomenon encoded in either SimLex or SimVerb (see Table 4). Overall, MEN is characterized by *higher* singular vectors, when SimLex and SimVerb are characterized by *lower* and more latent ones, which could explain the historical success of variance-based DSMs at capturing semantic relatedness rather than similarity. Moreover, our results show that models generated from different corpora will distribute information differently across their singular vectors, as shown per the variations of sampling patterns within identical similarity datasets displayed in Table 4: ACL-based DSMs for instance encode MEN much more latently in comparison to other corpora ($\bar{dim_{i}}=1,233\pm 43$) which explains the originally low performance on MEN of the variance-based DSM generated from ACL (see TOP scores for ACL in Table 3). In short, the information necessary to characterize a particular semantic phenomenon may actually be present (at least to some extent) in a given corpus, but not actually distributed in the top components of the SVD, calling once again into question the pertinence of the variance-preservation bias.

**TABLE 4:** Average mean, median and 90-th percentile of sampled dimensions indexes on MEN, SimLex and SimVerb for 10 shuffled runs in seq mode.

	MEN			SimLex			SimVerb		
	Median	Mean	90%	Median	Mean	90%	Median	Mean	90%
WIKI07	196 ± 12	576 ± 58	995 ± 237	612 ± 46	1,917 ± 107	6,314 ± 325	564 ± 45	1768 ± 98	6,227 ± 315
OANC	172 ± 9	567 ± 64	1,022 ± 168	677 ± 70	2,003 ± 92	6,499 ± 200	672 ± 68	2,210 ± 97	7,371 ± 200
WIKI2	220 ± 13	462 ± 48	917 ± 89	606 ± 35	1,218 ± 64	3,091 ± 242	586 ± 26	1,188 ± 60	2,847 ± 253
ACL	586 ± 15	1,233 ± 43	3,201 ± 178	935 ± 80	2,289 ± 106	7,376 ± 212	717 ± 47	1852 ± 79	6,012 ± 330
WIKI4	270 ± 11	532 ± 35	1,120 ± 59	662 ± 27	1,177 ± 50	2,635 ± 209	721 ± 37	1,297 ± 67	3,100 ± 260
BNC	163 ± 8	419 ± 48	651 ± 84	439 ± 22	969 ± 67	2,285 ± 291	518 ± 21	980 ± 41	2,254 ± 83

### 5.2 Better Superficial Alignment Does Not Mean Better Structural Alignment

**FIGURE 3:**
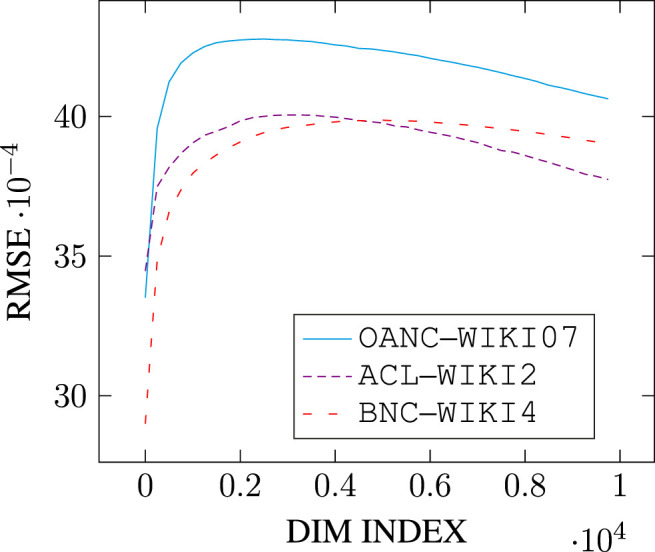
Evolution of RMSE for aligned bins of 250 consecutive singular vectors sampled across [0,10 000] for aligned corpora of different domains but similar size.

**FIGURE 4:**
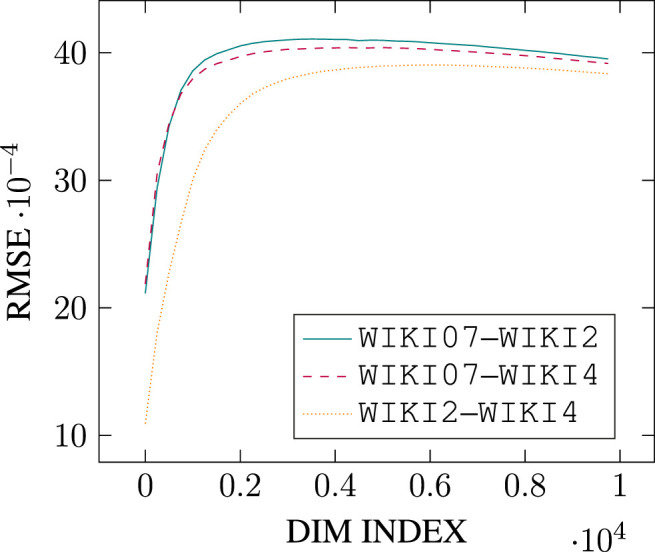
Evolution of RMSE for aligned bins of 250 consecutive singular vectors sampled across [0, 10 000] for aligned corpora of similar domains but different size.

Results of Section 5.1 show that explicit singular vectors sampling on MEN, SimLex and SimVerb leads to increased superficial alignment across datasets, and that the sampled singular vectors do *not* systematically correspond to the top components of the SVD. Still, would those specific sampling patterns also improve structural alignment between DSMs by lowering their RMSE? *Probably not*. To prove our point, let us plot the evolution of RMSE across bins of 250^14^ consecutive singular vectors, for corpora of same size but different domains (Figure 3) and different size but similar domains (Figure 4).

What those plots show first is the ability for our structural similarity metric to capture the intuition of *similar domains* across corpora: plots displaying the evolution of RMSE computed over pairs of models of partly overlapping Wikipedia samples follow much more similar trends than plots over pairs of models from different domains (compare gaps between plots across Figure 4 and Figure 3). What they show next, however, in that the RMSE is minimal for the top 250 components of the SVD and that it rapidly increases then. Therefore, any sampled set of 250 non-top singular vectors such as those reported in Table 4 will necessarily obtain a higher RMSE in comparison. In other words, increasing superficial alignment will necessarily decrease structural similarity.

### 5.3 Beyond Structural Alignment: Agreement vs. Compatibility

**FIGURE 5:**
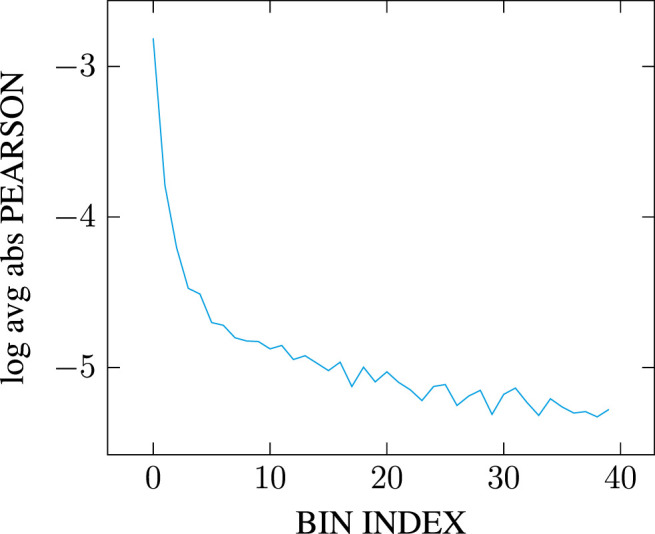
Evolution of the log of the average absolute pariwise Pearson correlation between singular vectors for bins of 250 sampled across [0,10 000] on OANC and WIKI07.

Figure 3 and Figure 4 exhibit a similar global pattern across aligned models: to minimize the RMSE, singular vectors can be sampled via the very top or the much more latent part of the SVD. Those two parts of the SVD, however, capture quite different information: more systematic information about language for the top components, and more idiosyncratic information regarding the corpus at hand for the more latent components. This phenomenon can be quantified by plotting the absolute Pearson correlation between pairs of singular vectors sampled across two DSMs (see Figure 5): top components have a correlation value closer to 1 ∼(log≈0) although it rapidly decreases as we move toward more latent singular vectors.

**FIGURE 6:**
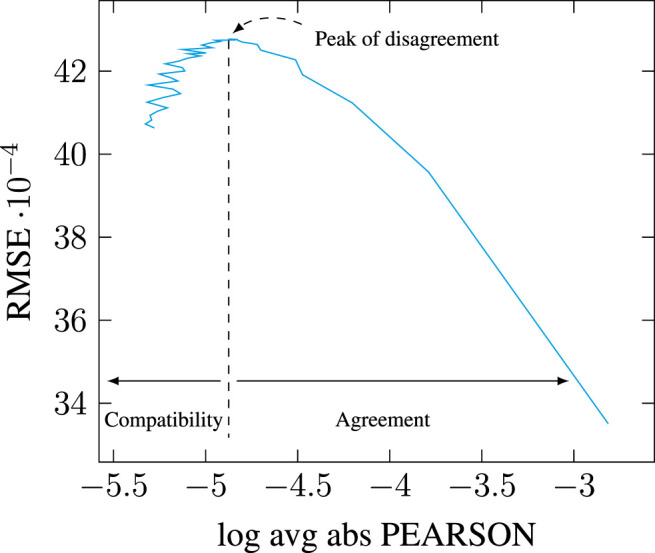
Evolution of RMSE with log of average absolute Pearson correlation for aligned bins of 250 consecutive singular vectors sampled across [0,10 000] on OANC and WIKI07.

And yet, as we plot the evolution of the RMSE as a function the Pearson correlation, averaged on bins of 30 consecutive singular vectors sampled across [0, 10 000], we do not observe a linear curve: that is, alignment does not get more and more difficult as the Pearson correlation decreases, but reaches a peak before significantly diminishing again (see Figure 6). This further illustrates a fundamental property of our alignment-based notion of similarity: two given models may be aligned if they both have *similar* components, but also if they have *dissimilar* components, provided that those components do not *conflict*. Notions of *agreement*, *compatibility* and *conflict* can be defined via the absolute Pearson correlation as described in Figure 6: maximal agreement is given by an absolute Pearson correlation of 1, and maximal compatibility is given by an absolute Pearson correlation of 0. In between, conflict increases as the absolute Pearson correlation goes down from full agreement to the peak of disagreement which maximizes the RMSE, then decreases again until it reaches maximal compatibility. Concretely, the peak of disagreement will correspond to sampling patterns that maximize structural dissimilarity between conceptual spaces, although this may not necessarily translate as superficial dissimilarity and explicit conflict between speakers during conversation, for reasons explained in Section 2.4. Note, moreover, that agreement and compatibility are defined on different domains: agreement is only defined rightward of the peak of disagreement, while compatibility is only defined leftward of the peak. Therefore, two speakers in full agreement cannot be said to have *incompatible* conceptual spaces.

A concrete example detailing the underlying mathematics of agreement and compatibility is given in Eq. 7: both matrix B and C can be aligned with matrix A when using our alignment algorithm, with a near-null RMSE ($<10^{-15}$). Yet, both matrices have quite different Pearson correlations: B’s and A’s elements have similar values and therefore A and B’s column vectors have a pairwise Pearson correlation of 1, while A and C’s pairwise Pearson correlation is merely at 0.3.

$$A=\left[\begin{array}{cccc} 1 & 0 & 0 & 0\\ 0 & 1 & 0 & 0\\ 0 & 0 & 1 & 0\\ 0 & 0 & 0 & 1 \end{array}\right]\;B=\left[\begin{array}{cccc} .9 & 0 & 0 & 0\\ 0 & .9 & 0 & 0\\ 0 & 0 & .9 & 0\\ 0 & 0 & 0 & .9 \end{array}\right]\;C=\left[\begin{array}{cccc} 0 & 0 & 0 & 1\\ 0 & 0 & 1 & 0\\ 1 & 0 & 0 & 0\\ 0 & 1 & 0 & 0 \end{array}\right]$$ (7)

This phenomenon directly relates to the “dog” example of Connell and Lynott (2014) previously detailed in Section 2.3, which showed how alignment may not always equate agreement but sometimes mere compatibility between conceptual representations: speakers holding marginally identical conceptual representations—in this case widely differing representations of prototypical dogs *size*—can still be assumed to understand one-another, especially if disagreement pertains to aspects of conceptual knowledge that are irrelevant to the conversation at hand. Our experimental results support the idea that such considerations also extend to conceptual *spaces* and notions of structural similarity: widely differing aggregates of contextual experience captured by singular vectors can still sometimes provide a solid basis for structural alignment. Our characterizations of notions of structural agreement and compatibility, however, are more flexible than previous ones, in that they notably do not require a form of explicit, lexicalized, “feature-based” interpretation of what they entail. In our case, they can be defined in a more systematic fashion as a form of latent structural property of the conceptual space with respect to alignment.

## 6. Discussion

### 6.1. Why Is Compatibility Relevant Anyway?

Why should we care about compatibility in the first place? After all, Figures 3, 4, and 6 combined show that the RMSE is significantly lower in the agreement zone than in the compatibility zone, especially for the top components of the SVD. Why should speakers striving to align their conceptual spaces, then, not end up sampling those top components, and only those top components? The answer to that question will depend on how many singular vectors we can reasonably assume to be sampled during a realistic coordination setting. Because the RMSE is certainly lowest for the top components of the SVD, but those top components are actually not that many: after the first 250 singular vectors, the RMSE then significantly increases across all corpora in a systematic fashion.

And indeed when looking at it more closely, the compatibility zone appears to include *many more* singular vectors than the agreement zone. Our results show indeed that the peak of disagreement is located roughly at d = 2,175 for OANC–WIKI07, d=2,850 for ACL–WIKI2, and d=4,750 for BNC–WIKI4, out of 10,000 singular vectors in total. Yet the comparison does not stop there as the location of the peak of disagreement alone does not guarantee that singular vectors sampled from the agreement zone will systematically lead to lower RMSE compared to singular vectors sampled from the compatibility zone. As a matter of fact, numbers drop even further then: only about 225 singular vectors of the 2,175 that are in the agreement zone of OANC–WIKI07 can lead to a lower RMSE than the lowest RMSE of the compatibility zone. For ACL–WIKI2, the corresponding number is about 250 out of 2,850, and for BNC–WIKI4, 1,400 out of 4,750.^15^

Concretely, what those results suggest is that every ad-hoc coordination scenario characterized by a sampling pattern comprising more than 225, 250 and 1,400 vectors respectively will have to select singular vectors in the compatibility zone in order to minimize the RMSE. And there is every reason to expect that the order of magnitude of the number of vectors sampled during a realistic coordination scenario will be even higher than that. SimVerb, on that matter, may provide an interesting perspective, as it almost systematically leads to larger sampled sets of singular vectors: closer to 300 average, while MEN and SimLex remain at 200 (see Table 3). One could assume first such differences to constitute byproducts of the number of constraints encoded by each dataset: SimVerb is indeed supposed to characterize the same notion of *similarity* than SimLex but does so on a much larger sample of word pairs (3,500 vs. 999). Yet, *quantity* may not be the sole key factor here, as MEN also characterizes constraints on about 3,000 word pairs, with a similar sampling average than SimLex.

The *quality* and *nature* of those constraints may prove more determinant indeed: SimVerb encodes more fine-grained nuances on a much narrower conceptual domain in comparison to the other datasets, which could explain why it actually requires additional singular vectors to be characterized. Furthermore, we will argue here that the nature of its constraints probably makes SimVerb a much more adequate and representative lexical similarity dataset for the task at hand. Coordination, we would argue, is indeed probably better approximated by the idea that speakers align their similarity judgments on verbs like *enforce* and *impose*, rather than on the fact that *automobile* and *car* should be deemed related while *dog* and *silver* should not, as in MEN, or on the fact that *arm* and *shoulder* should be deemed similar, while *hard* and *easy* should not, as in SimLex.

If our intuition is correct, then maybe what we need in computational linguistics to better model coordination are lexical similarity datasets that encode very nuanced distinctions between lexical items, rather than broad semantic categorizations. In any case, it does not seems completely unreasonable to assume that, in a realistic ad-hoc coordination scenario, sampled vectors will ultimately fall into the compatibility zone in order to minimize the RMSE. All in all, compatibility should matter then in order to optimize structural conceptual alignment.

### 6.2. Compatibility Emerges From Idiosyncrasy

Considering it plausible for singular vectors to be sampled from the compatibility zone is one thing, but it does not tell us *how many of them* will actually be sampled. In order to make a point about the significance of the compatibility phenomenon, we must first indeed guarantee that the number of vectors sampled from the compatibility zone will not be marginal in comparison to the agreement zone. Is the size of the compatibility zone reported in Section 6.1, then, a reasonable approximation of the reality or a mere artifact of our experimental setup?

To answer this question, we must first understand where this compatibility phenomenon comes from. Recall from Section 5.3 that the compatibility zone corresponds to the lower components of the SVD which capture more idiosyncratic information regarding the corpus at hand, in comparison to the top components which capture more systematic information about language. Agreement and compatibility are therefore first and foremost characterized by different distributional patterns across corpora, themselves deriving from differences over co-occurrence *counts*. Indeed, count-based DSMs only aggregate information from word-context co-occurrences, so that differences across aggregated distributional patterns are necessarily byproducts of cascading differences originating from the raw count matrices (recall Figure 2).

Yet, this particular focus on co-occurrence counts glosses over an important modeling choice of ours: in our experimental setup, DSM vocabularies are aligned *after* the SVD step, and not *before*. Therefore, the raw count matrix of a particular DSM may aggregate information over context words that are absent from other DSMs. In effect, this is tantamount to assuming that different speakers could process external stimuli from a different set of cognitive receptors, or that they could process external stimuli from a shared set of cognitive receptors but that some of those receptors will only be triggered in specific speakers.

And how much would the set of receptors differ across speakers then? Pretty much, according to our results: for the OANC–WIKI07 pair for instance, 36% of the words in OANC are not found in WIKI07, while 62% of the words in WIKI07 are not found in OANC. Note, however, that due to the Zipfian distribution of words in each corpus (Zipf, 1936; Zipf, 1949) those out-of-shared-vocabulary words only account for 2% and 3% of the total corpus word counts respectively.

**FIGURE 7:**
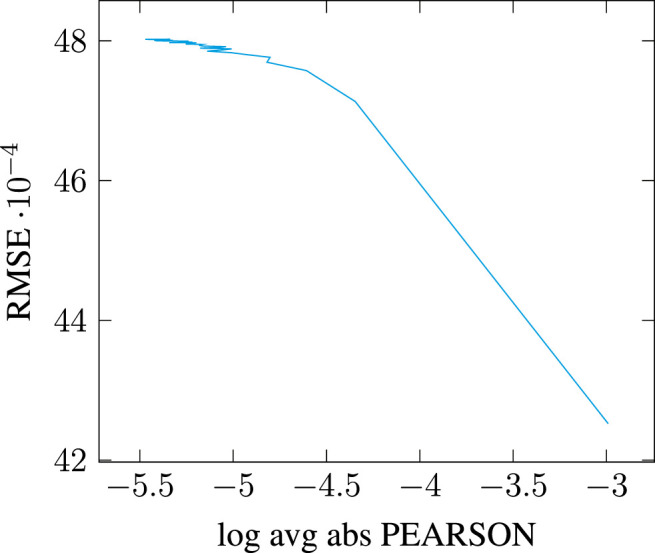
Evolution of RMSE with log of average absolute Pearson correlation for aligned bins of 250 consecutive singular vectors sampled across [0,10 000] on OANC and WIKI07, for DSMs with vocabularies aligned *before* the SVD step.

What happens, then, if we align vocabularies across DSMs *before* the SVD step and filter out context columns of the original raw count matrices for words outside of the shared vocabulary? Our results, displayed in Figure 7, show that *the phenomenon of compatibility almost completely disappears*.

Those results have fundamental consequences for the socialization hypothesis. Indeed, they show that, if differences across speakers’ background experiences are to be understood as differences in distributional patterns over external stimuli triggering a shared set of cognitive receptors, then in fact *alignment equates agreement* so that it should indeed be impossible for speakers to coordinate and align their respective conceptual spaces if those are grounded in fundamentally different background experiences.

Of course the aforementioned considerations could be deemed artifactual of the SVD and more specifically of its sensitivity to null values in the original PPMI matrix: Landauer and Dumais (1997), for instance, already noted that “a change in the value of any cell in the original matrix can, and usually does, change every coefficient in every condensed word vector” (see p. 218), while Levy and Goldberg (2014), citing (Koren et al., 2009), stressed how SVD is known to suffer from unobserved values (see p. 6). But this would only provide a technical explanation while the main question remains: should we consider this artifact to be present in human cognition as well? Probably so, at least if we are to consider conceptual knowledge to emerge from contingency-based aggregation and covariation-based decomposition of distributional information (see Section 3.3.3).

ll in all, our results show that *compatibility emerges from idiosyncrasy*, but that idiosyncracy here should not be understood as a distinctive difference in the *distribution* of information across background experiences, but as a difference of *nature*. Compatibility, so it seems, emerges from the *uniqueness* of each speaker and from aspects of their background experiences that uniquely distinguish them from others. Coordination, then, is enabled by what makes speakers *unique* rather than *different* from one-another.

Yet, is it completely realistic to consider that the background experience of a speaker could be primarily constituted (for more than 60% as our results above suggest) of stimulus components not experienced at all by other speakers, even if those stimuli account for a tiny portion of the overall experienced stimuli? Interestingly, those considerations directly connect us with the longstanding debate in cognitive science regarding the nature of conceptual knowledge. The fundamental question, as Huebner and Willits (2018) frame it, is really whether “knowledge consists primarily (or exclusively) of a rich sets of associations between sensory-motor features, or instead also consists of abstract, amodal concepts that bind those features together”. For if indeed conceptual knowledge is to be aggregated mostly from sensorimotor experience, it seems dubious to consider contextual vectors in DSMs to model anything but low-level core cognitive components, necessarily shared across speakers. All the more so if we are to follow previous approaches detailed in Section 3.3.1 and consider distributional linguistic information to mirror distributional information grounded in sensorimotor experience.

But if, however, we are to consider conceptual knowledge to be aggregated mostly from *pre-existing* intermediate conceptual knowledge, a new perspective opens. Most concepts become *complex* concepts, and DSMs now model distributional learning mediated by a speaker-specific intermediate cognitive layer, rather than a set of universal core cognitive components. An unexpected solution to our puzzle appears to rest on the possible compromise between two seemingly incompatible approaches to human cognition.

## 7. Conclusion

Do speakers of the same linguistic community share similar concepts given that they are exposed to similar environments and operate in highly-coordinated social contexts? In as much as the notion of *similarity* hereby specified entails *agreement* between speakers and their conceptual spaces, the claim remains to be proven, for non-trivial conceptual variability between speakers systematically observed across experimental setups continues to be a major obstacle to be accounted for.

Yet, if we are to distinguish within similarity the notion of *agreement* from that of *compatibility*, new perspectives open: speakers no longer need to converge to *close-enough* conceptual representations in order to successfully communicate, for agreement is no longer necessary when you can merely *avoid conflict* by aligning your non-identical but nonetheless compatible representations. Even more so as this notion of compatibility leaves ample room for adjustments across speakers and thus, ultimately, successful coordination and communication. From latent compatibility to superficial agreement: all we need is a tiny conceptual shift in our characterization of similarity.

Although the cognitive plausibility of our proposed model remains to be assessed, it already provides an intuitive explanation to the very problem of conceptual variability, henceforth conceived as a mere artifact of conceptual compatibility. Indeed, our experimental approach shows that the number of compatible subspaces largely extend the number of agreeing ones, so that speakers can never be expected to agree more than to some extent. Conceptual variability should therefore not be seen as a byproduct of faulty experimental setups, but rather as a key property of human cognition.

All in all, the socialization hypothesis may very well prove to be an unnecessary prerequisite to successful communication. But our study suggests implicitly that other assumptions grouding standard models of communication could also prove unnecessary, if not unfounded. The *identicity of messages*, assumed to characterize communication success in a standard Shannon–Weaver code model, could be one of them.

All things considered indeed, communication may probably be best formalized as the cooperative act of *avoiding conflict*, rather than maximizing agreement.

## Data Availability Statement

All data and softwares used throughout this work can be found at https://gitlab.com/akb89/avoiding-conflict

## Author Contributions

AK came up with the original idea, designed and carried out the experiments. AH supervised the work. Both authors contributed to the writing of the paper.

## Conflict of Interest

The authors declare that the research was conducted in the absence of any commercial or financial relationships that could be construed as a potential conflict of interest.

## Footnotes

1 For earlier work on lexical coordination focusing on reference, see (Clark and Wilkes-Gibbs, 1986; Brennan and Clark, 1996).

2 The role of coordination in the socialization hypothesis is explicit in Barsalou’s characterization introduced in Section 2.1.

3 In case readers were to wonder whether her experimental protocol were not forcing artificial similarity judgments upon subjects, note that Gentner (1988) specifically mentions cases were children explicitly reject metaphorical interpretations for concepts they do not consider to be similar.

4 We further discuss the influence of the *α* parameter in Section 5.1.

5 See also (Osgood, 1952, p. 228) very similar in spirit.

6 See also (Saffran, 2003; Smith and Yu, 2008; Aslin and Newport, 2012; Hall et al., 2018).

7 Although see maybe (Papini and Bitterman, 1990) for a counter-argument.

8 Episodic memory is assumed to contain memory of autobiographical events while semantic memory is assumed to be dedicated to generalized memory not linked to specific events (Tulving, 1972).

9 We are not arguing here that similarity judgments are always necessarily “feature-based” or that there could exist more prominent features systematically influencing similarity judgments. We only provide this example for illustrative purposes in order to give the reader a better intuition of our sampling algorithm’s underlying logic. Nonetheless, our examples remains grounded in empirical evidence which show, e.g., that novices tend to judge similarity based on superficial or surface features, whereas experts rely on deeper underlying principles (Chi et al., 1981). So in fact our example is not completely implausible as differences in knowledge grounded in differences across background experiences could perfectly translate as differences in similarity judgments: a biologist could be more inclined to consider that Cat is more similar to Tiger than to Dog on the ground of their being both part of the *felidae* family, while someone else, say a child, unaware of this sort of classification, could consider Dog and Cat to be more similar on the basis of their being both of similar *shape* or *size*.

10 Our choice of k=10 000 is questionable given that we previously showed in Table 1 that it could at best retain 72% of the total energy. It is primarily motivated by computational considerations and the necessity to maintain acceptable overall computing time. As we show in Section 5.1 it appears to be a reasonable compromise given than 90% of the sampled dimensions on all our DSMs and across all our lexical similarity datasets remain below the 8,000th dimension.

11 Note that in order to minimize interferences with reported results, we perform pre-validation on the MEN and SimLex datasets only, using DSMs generated exclusively from the WIKI corpus. Full details regarding this point are made available in the Supplementary Material.

12 On practical matters, note that a rich literature exists on incremental SVD update (e.g., Businger, 1970; Bunch and Nielsen, 1978) so that our particular implementation would not necessarily constitute an obstacle to modeling online coordination: see (Gentle, 2009) for a comprehensive introduction to the topic. Brand (2003, 2006) has notably proposed an algorithm for incrementally adding, removing and updating rows and columns in the SVD matrix that could prove particularly useful for that purpose.

13 As implemented by Philipp Stinger: https://github.com/psinger/CorrelationStats/blob/master/corrstats.py

14 Corresponding to the rough average number of singular vectors sampled across models and datasets in Table 3.

15 All those numbers were computed for small bins of 25 singular vectors to get a more fine-grained appreciation of the evolution of the RMSE across the SVD spectrum.
