# Supplementary material for: Avoiding Conflict: When Speaker Coordination Does Not Require Conceptual Agreement
Source: Front Artif Intell. 2021 Jan 27;3:523920. doi: 10.3389/frai.2020.523920 (PMC7861244; doi:10.3389/frai.2020.523920)
Supplement: Supplementary file 1 [file datasheet1.pdf]

Supplementary Material

1 SUPPLEMENTARY EXPERIMENTAL SETUP

This section details the pre-validation step used to select the best optimization metric to be used on train folds by our sampling algorithm. We tested four possible metrics:

1. Spearman correlation (spr), which only assesses the *rank* correlation between DSMs pairwise similarity scores and those of the lexical similarity dataset;
2. Pearson correlation (pearson), which is the standard bivariate correlation, supposedly more ‘constraining’ than the Spearman correlation as it also takes into account the correlation between the absolute similarity scores;
3. Root Mean Square Error (rmse), which is computed on the two sets of absolute similarity scores generated by the DSM and the lexical similarity dataset, supposedly even more constraining than Pearson correlation as it assesses the proximity between absolute similarity scores and not only their correlation; and
4. Spearman and Root Mean Square Error jointly (spr+rmse), which should provide an intermediate constraint between bare spr and bare rmse.

Note that, in order to minimize interferences with our general experimental setup and guarantee the robusteness of our reported results, we perform pre-validation on the `MEN` and `SimLex` datasets only (keeping `SimVerb` for validation) using DSMs generated exclusively from the `WIKI` corpus. Note also that scores on test folds always report the Spearman correlation, as per the standard evaluation setup on lexical similarity datasets.

Our results, detailed in Table S1 below, show that overall bare Spearman and Pearson correlations are too easy constraints that lead to significant overfitting, as measured per the difference between train and test folds Spearman correlations. What this means is that the sampling algorithm can select many singular vectors that will actually increase the Spearman correlation with the train folds of the corresponding lexical similarity dataset, but that those improvements will not carry over to the test folds.

RMSE provides the most robust constraint with the lowest overfitting, but at the cost of lower absolute test scores. Overall, jointly optimizing both the RMSE and the Spearman correlation appears to be the best compromise, with relatively low overfitting and best test scores.

	dataset	spr	pearson	rmse	spr+rmse
train-test spr diff	`MEN`	0.26 ± 0.00	0.20 ± 0.00	**0.02** $\pm$ 0.00	0.04 ± 0.00
train-test spr diff	`SimLex`	0.52 ± 0.01	0.58 ± 0.01	**0.19** $\pm$ 0.01	0.26 ± 0.01
test spr	`MEN`	0.65 ± 0.00	0.68 ± 0.00	0.66 ± 0.00	**0.74** $\pm$ 0.00
test spr	`SimLex`	0.36 ± 0.01	0.34 ± 0.01	0.34 ± 0.01	**0.43** $\pm$ 0.01

Table S1. Quantifying overfitting in `seq` sampling for various optimization metrics: Spearman correlation (spr), Pearson correlation (pearson), Root Mean Square Error (rmse) or Spearman correlation and Root Mean Square Error jointly (spr+rmse). *train-test spr diff* reports absolute differences of Spearman correlation between train and test folds using 5-fold validation and reporting averaged test scores with the standard error. *test spr* reports averaged Spearman correlations on test folds. All models are PPMI-weighted count-based DSMs generated from the `WIKI` corpus with a window size of 2.

2 SUPPLEMENTARY RESULTS

2.1 Effects of window size are amplified by the variance-preservation bias

The results displayed in Section 5.1 show that the information one needs to characterize a given semantic phenomenon may actually be present in the original SVD matrix, but that it may be missed if sampling only the top singular vectors. The question we wanted to ask, then, was whether calling into question the variance-preservation bias did not have cascading consequences on other historical features of DSMs. In this section we focus on the *window size* hyperparameter and show that its influence on the ability of DSMs to capture specific semantic phenomena can actually be considered somehow artifactual of the variance-preservation bias itself.

The window size hyperarameter has indeed long been argued to directly influence the performance of DSMs: Bullinaria and Levy (2007) originally stated that small windows were only appropriate for syntactic tasks and larger ones for semantic tasks, with no clear optimal window size overall for all tasks (see p.518). Turney (2012) further argued that larger windows captured the topic or domain of a word, while smaller windows emphasized word function (see also Agirre et al., 2009; Kiela and Clark, 2014; Levy and Goldberg, 2014; Levy et al., 2015; Chiu et al., 2016). Hill et al. (2015) finally argued that small context windows better captured *similarity* while larger ones better captured *relatedness*, an observation later confirmed in (Lison and Kutuzov, 2017, p.286), although Hill et al. (2015) nuanced their original claim by arguing that the ability for small context windows to capture similarity depended (in part) on the general DSM architecture (see p.691).

However, if the window size parameter leads to information being spread differently across singular vectors, we may wonder whether the aforementioned observations do not only hold when the window size parameter is considered *jointly* with the variance-preservation bias. Once again, information may be spread differently across the SVD but nonetheless present albeit not in the top components of the matrix. Thus, our `seq` sampling algorithm could potentially prove capable of mitigating the differences observed across DSMs generated with different window sizes.

Our results on the matter are mixed, but show nonetheless that effects of window size are indeed amplified by the variance-preservation bias (see Table S2). Mixed, first of all, because not all differences observed across TOP models of different window size appear to be statistically significant in the first place. Indeed, across 20 pairs of DSMs with window size in $[1, 2, 5, 10, 15]$, 15 out of 20 appear to exhibit statistically significant differences on `MEN`, but only 4 out of 20 on `SimLex` and 3 out of 20 on `SimVerb`. This relates to a broader issue in computational linguistics at large, in that very few research actually did originally report the statistical significance of the difference of performance observed on lexical similarity (see, e.g. Rastogi et al., 2015; Faruqui et al., 2016; Dror et al., 2018). Anyhow, the number of statistically significant differences *does* reduce with SEQ models, although it does not completely disappear: from 15 to 8 statistically significant differences on `MEN`, it drops from 4 to 0 on `SimLex` and remains at 3 on `SimVerb`, albeit on a different set of model pairs (compare TOP and SEQ models on `SimVerb` in Table S2).

All in all, larger context windows in PPMI-weighted count-based DSMs only appear to better capture relatedness *to some extent*. Indeed, this effect appears heavily tied to the variance-preservation bias, since almost half of the statistically significant differences vanish once the bias is removed. Else, and contrary to previous studies, our results do not provide support for the claim that smaller context windows better capture similarity, as all window sizes appear to perform equally well on both `SimLex` and `SimVerb`.

	win-1	win-2	win-5	win-10	win-15
			TOP models on MEN		
win-1	-	**0.0006**	**$< 10^{-4}$**	**$< 10^{-4}$**	**$< 10^{-4}$**
win-2	**$< 10^{-4}$**	-	0.0605	0.1590	0.3257
win-5	**$< 10^{-4}$**	**$< 10^{-4}$**	-	**0.0099**	0.0490
win-10	**$< 10^{-4}$**	**$< 10^{-4}$**	**$< 10^{-4}$**	-	**0.0004**
win-15	**$< 10^{-4}$**	0.0125	**0.0003**	**$< 10^{-4}$**	-
			SEQ models on MEN		
win-1	-	0.2724	**0.0022**	**0.0038**	**0.0008**
win-2	0.0664	-	0.1045	0.0226	0.2639
win-5	**$< 10^{-4}$**	**0.0001**	-	0.3320	0.0242
win-10	**$< 10^{-4}$**	**0.0061**	0.0116	-	0.0276
win-15	**0.0002**	0.0197	0.3614	0.4820	-
			TOP models on SimLex		
win-1	-	0.1409	0.5412	0.4255	0.2190
win-2	0.0516	-	0.0952	0.0191	0.0633
win-5	0.2530	0.2200	-	0.0296	**0.0042**
win-10	0.6356	0.4415	0.4671	-	0.0590
win-15	0.0227	**0.0069**	**$< 10^{-4}$**	**$< 10^{-4}$**	-
			SEQ models on SimLex		
win-1	-	0.0973	0.0373	0.1289	0.0157
win-2	0.5787	-	0.0108	0.2287	0.0566
win-5	0.3720	0.5468	-	0.1946	0.1228
win-10	0.3306	0.4569	0.4176	-	0.1207
win-15	0.1953	0.3575	0.2768	0.0490	-
			TOP models on SimVerb		
win-1	-	**0.0007**	0.0192	0.1169	0.0668
win-2	**0.0022**	-	0.4289	0.2807	0.2572
win-5	**0.0008**	0.1658	-	0.1811	0.2252
win-10	0.0188	0.3716	0.1237	-	0.0604
win-15	0.0668	0.2849	0.0820	0.1930	-
			SEQ models on SimVerb		
win-1	-	0.0518	0.5489	0.3151	0.4053
win-2	0.0129	-	0.1690	0.0594	**0.0017**
win-5	0.0649	0.2534	-	0.1411	**0.0051**
win-10	0.1559	0.1340	0.0832	-	**0.0050**
win-15	0.4053	0.1251	0.5678	0.1750	-

Table S2. Statistical significance (p values) of Spearman correlations differences computed on MEN, SimLex and SimVerb for DSM pairs of PPMI-weighed count-based models generated from the full Wikipedia (WIKI) corpus with various window sizes. SEQ models are reduced via our seq algorithm detailed in Section 4.1, while TOP models are reduced by selecting the top $n = ndim$ singular vectors from the SVD matrix, with $ndim$ the number of dimensions sampled by the corresponding SEQ model. Horizontal lines indicate either best SEQ performing models for each window size, taken after 10 shuffled run, either corresponding TOP models. Vertical lines indicate, for each horizontal line, the equivalent model (same dimensionality and same kfold validation) for the corresponding window size. Bold values indicate statistically significant differences ($p < .01$).

Else, the contribution of our seq algorithm also carries over to models of different window sizes: Table S3 shows that replacing the traditional variance-preservation bias with our sampling algorithm leads

to near-systematic improvements on DSMs generated from the `WIKI` corpus with different window sizes, and that on all corpora.

model	α	win-1	win-2	win-5	win-10	win-15
				`MEN`		
TOP	0	0.67 ± 0.01	0.71 ± 0.00	0.72 ± 0.01	0.72 ± 0.02	0.73 ± 0.0
SEQ	-	**0.74** $\pm$ 0.01	**0.75** $\pm$ 0.01	**0.77** $\pm$ 0.01	**0.77** $\pm$ 0.01	**0.77** $\pm$ 0.0
p		$< 10^{-4}$	$< 10^{-4}$	$< 10^{-4}$	$< 10^{-4}$	$< 10^{-4}$
ndim		279 ± 11	268 ± 7	181 ± 3	182 ± 7	189 ± 9
				`SimLex`		
TOP	0	0.30 ± 0.02	0.34 ± 0.02	0.32 ± 0.02	0.31 ± 0.03	0.29 ± 0.02
SEQ	-	**0.47** $\pm$ 0.03	**0.46** $\pm$ 0.02	0.41 ± 0.02	0.42 ± 0.02	**0.38** $\pm$ 0.03
p		$< 10^{-4}$	0.0015	0.0114	0.0145	0.0013
ndim		195 ± 4	235 ± 7	250 ± 12	257 ± 11	213 ± 9
				`SimVerb`		
TOP	0	0.16 ± 0.02	0.22 ± 0.02	0.21 ± 0.01	0.22 ± 0.02	0.20 ± 0.02
SEQ	-	**0.32** $\pm$ 0.02	**0.36** $\pm$ 0.01	**0.34** $\pm$ 0.01	**0.33** $\pm$ 0.03	**0.31** $\pm$ 0.02
p		$< 10^{-4}$	$< 10^{-4}$	$< 10^{-4}$	$< 10^{-4}$	$< 10^{-4}$
ndim		405 ± 19	344 ± 17	303 ± 9	389 ± 16	386 ± 7

Table S3. Spearman correlations on `MEN`, `SimLex` and `SimVerb` for DSMs generated from the `WIKI` corpus with different window sizes. SEQ models are reduced via our `seq` algorithm while TOP models are reduced by selecting the top $n = ndim$ singular vectors from the SVD matrix, with $ndim$ corresponding for each fold to the number of dimensions sampled by the SEQ model on that fold. All results are averaged across test folds applying 5-fold validation, after taking the best of 10 shuffled runs. Bold results indicate statistically significant differences ($p < .01$) between SEQ and TOP models.

In addition, Table S4 shows that different window sizes lead to differences in the spread of distributional information across the singular vectors of the SVD matrix so that, once again, characterizing different semantic phenomena will lead to different sampling patterns across dimensions.

	`MEN`			`SimLex`			`SimVerb`		
	median	mean	90%	median	mean	90%	median	mean	90%
win-1	342 ± 19	610 ± 39	1434 ± 80	794 ± 29	1236 ± 55	2801 ± 126	1197 ± 32	1519 ± 38	3126 ± 85
win-2	244 ± 17	518 ± 36	1252 ± 98	944 ± 32	1461 ± 63	3390 ± 148	960 ± 33	1265 ± 40	2677 ± 89
win-5	172 ± 6	413 ± 29	899 ± 50	812 ± 47	1266 ± 40	2980 ± 99	1037 ± 31	1391 ± 44	2813 ± 97
win-10	185 ± 8	483 ± 31	1110 ± 117	808 ± 30	1357 ± 46	3288 ± 131	1034 ± 33	1441 ± 43	3019 ± 117
win-15	221 ± 12	533 ± 31	1155 ± 100	718 ± 53	1237 ± 79	3102 ± 224	978 ± 29	1434 ± 41	3169 ± 135

Table S4. Average mean, median and 90-th percentile of sampled dimensions indexes on `MEN`, `SimLex` and `SimVerb` for 10 shuffled runs in `seq` mode.

2.2 Agreement and compatibility on `ACL`, `WIKI2`, `BNC` and `WIKI4`

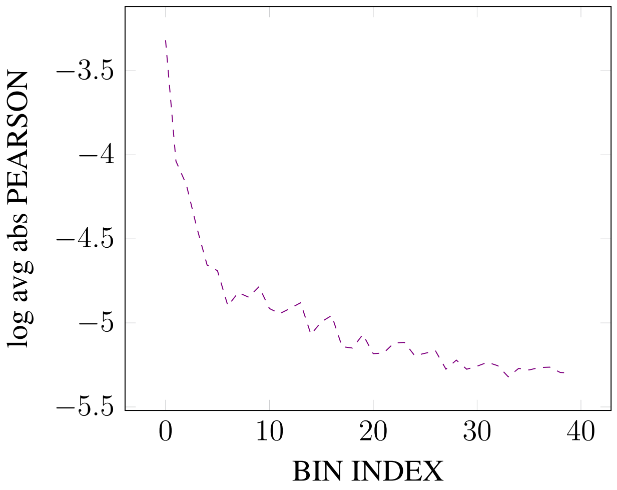

Figure S1: Evolution of the log of the average absolute pairwise Pearson correlation between singular vectors for bins of 250 sampled across $[0, 10\,000]$ on `ACL` and `WIKI2`.

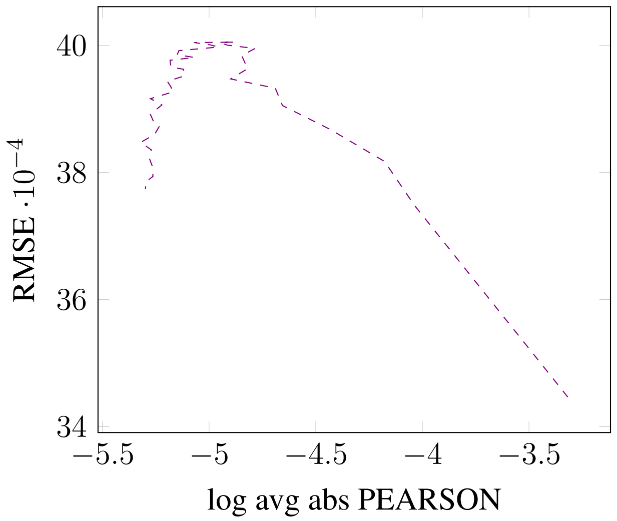

Figure S2: Evolution of RMSE with log of average absolute Pearson correlation for bins of 250 consecutive singular vectors sampled across $[0, 10\,000]$ on `ACL` and `WIKI2`.

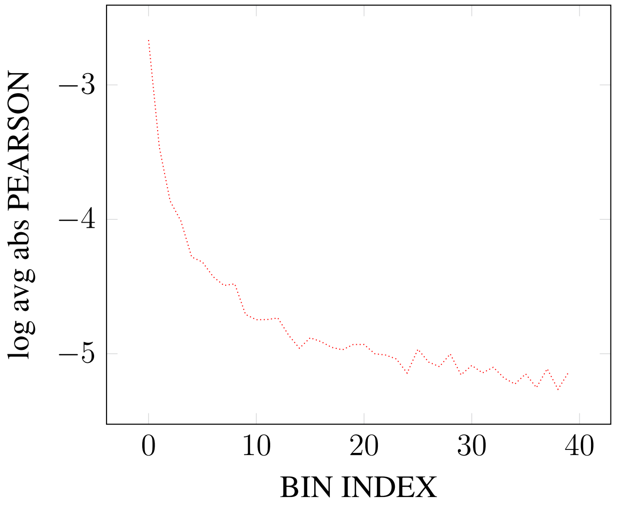

Figure S3: Evolution of the log of the average absolute pairwise Pearson correlation between singular vectors for bins of 250 sampled across $[0, 10\,000]$ on `BNC` and `WIKI4`.

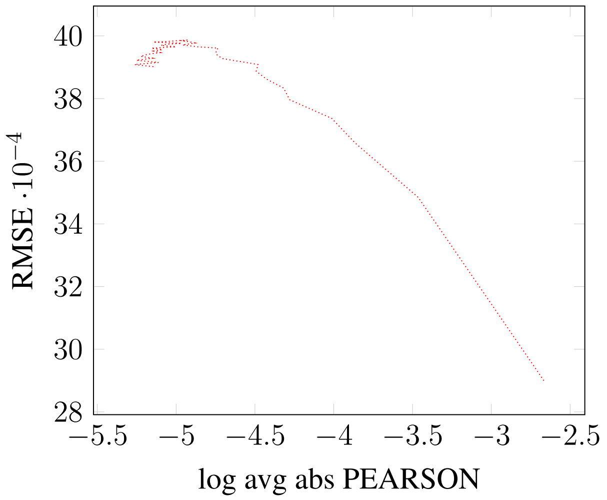

Figure S4: Evolution of RMSE with log of average absolute Pearson correlation for bins of 250 consecutive singular vectors sampled across $[0, 10\,000]$ on `BNC` and `WIKI4`.

3 SUPPLEMENTARY DISCUSSION

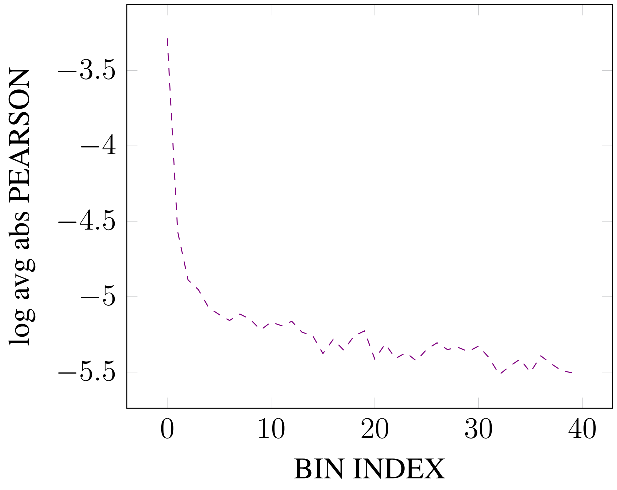

Figure S5: Evolution of the log of the average absolute pairwise Pearson correlation between singular vectors for bins of 250 sampled across $[0, 10\,000]$ on `ACL` and `WIKI2`, for DSMs with vocabularies aligned *before* the SVD step.

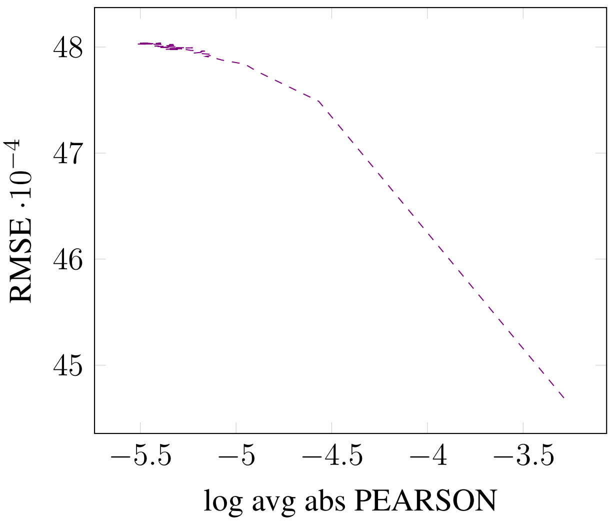

Figure S6: Evolution of RMSE with log of average absolute Pearson correlation for bins of 250 consecutive singular vectors sampled across $[0, 10\,000]$ on `ACL` and `WIKI2`, for DSMs with vocabularies aligned *before* the SVD step.

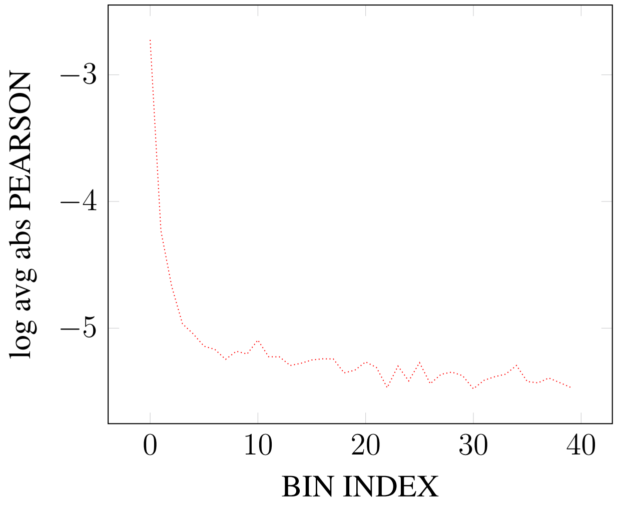

Figure S7: Evolution of the log of the average absolute pairwise Pearson correlation between singular vectors for bins of 250 sampled across $[0, 10\,000]$ on `BNC` and `WIKI4`, for DSMs with vocabularies aligned *before* the SVD step.

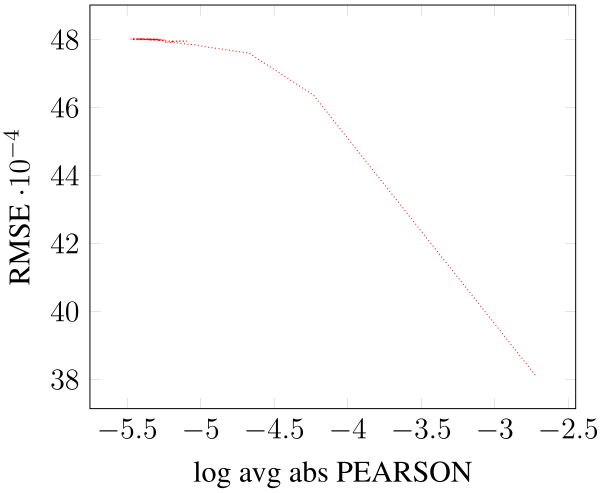

Figure S8: Evolution of RMSE with log of average absolute Pearson correlation for bins of 250 consecutive singular vectors sampled across $[0, 10\,000]$ on `BNC` and `WIKI4`, for DSMs with vocabularies aligned *before* the SVD step.

REFERENCES

Agirre, E., Alfonseca, E., Hall, K., Kravalova, J., Pasca, M., and Soroa, A. (2009). A study on similarity and relatedness using distributional and wordnet-based approaches. In *Proceedings of Human Language Technologies: The 2009 Annual Conference of the North American Chapter of the Association for Computational Linguistics* (Association for Computational Linguistics), 19–27

Bullinaria, J. A. and Levy, J. P. (2007). Extracting semantic representations from word co-occurrence statistics: A computational study. *Behavior research methods* 39, 510–526

Chiu, B., Korhonen, A., and Pyysalo, S. (2016). Intrinsic evaluation of word vectors fails to predict extrinsic performance. In *Proceedings of the 1st Workshop on Evaluating Vector-Space Representations for NLP* (Association for Computational Linguistics), 1–6. doi:10.18653/v1/W16-2501

Dror, R., Baumer, G., Shlomov, S., and Reichart, R. (2018). The hitchhiker's guide to testing statistical significance in natural language processing. In *Proceedings of the 56th Annual Meeting of the Association for Computational Linguistics (Volume 1: Long Papers)* (Melbourne, Australia: Association for Computational Linguistics), 1383–1392. doi:10.18653/v1/P18-1128

Faruqui, M., Tsvetkov, Y., Rastogi, P., and Dyer, C. (2016). Problems with evaluation of word embeddings using word similarity tasks. In *Proceedings of the 1st Workshop on Evaluating Vector-Space Representations for NLP* (Association for Computational Linguistics), 30–35. doi:10.18653/v1/W16-2506

Hill, F., Reichart, R., and Korhonen, A. (2015). Simlex-999: Evaluating Semantic Models with Genuine Similarity Estimation. *Computational Linguistics* 41, 665–695. doi:10.1162/COLI_a_00237

Kiela, D. and Clark, S. (2014). A systematic study of semantic vector space model parameters. In *Proceedings of the 2nd Workshop on Continuous Vector Space Models and their Compositionality (CVSC)* (Association for Computational Linguistics), 21–30. doi:10.3115/v1/W14-1503

Levy, O. and Goldberg, Y. (2014). Dependency-Based Word Embeddings. In *Proceedings of the 52nd Annual Meeting of the Association for Computational Linguistics (Volume 2: Short Papers)* (Baltimore, Maryland: Association for Computational Linguistics), 302–308

Levy, O., Goldberg, Y., and Dagan, I. (2015). Improving distributional similarity with lessons learned from word embeddings. *Transactions of the Association for Computational Linguistics* 3, 211–225

Lison, P. and Kutuzov, A. (2017). Redefining context windows for word embedding models: An experimental study. In *Proceedings of the 21st Nordic Conference on Computational Linguistics* (Gothenburg, Sweden: Association for Computational Linguistics), 284–288

Rastogi, P., Van Durme, B., and Arora, R. (2015). Multiview LSA: Representation learning via generalized CCA. In *Proceedings of the 2015 Conference of the North American Chapter of the Association for Computational Linguistics: Human Language Technologies* (Denver, Colorado: Association for Computational Linguistics), 556–566. doi:10.3115/v1/N15-1058

Turney, P. D. (2012). Domain and function: A dual-space model of semantic relations and compositions. *Journal of Artificial Intelligence Research* 44, 533–585
